# The DREAMS START intervention for sleep in dementia: Long‐term follow‐up of a randomized controlled trial

**DOI:** 10.1002/alz.71274

**Published:** 2026-03-11

**Authors:** Penny Rapaport, Mariam O. Adeleke, Julie A. Barber, Lina Gonzalez, Rachael Hunter, Monica Manela, Sarah Amador, Sube Banerjee, Georgina Charlesworth, Chris Clarke, Colin A. Espie, Simon D. Kyle, Malgorzata Raczek, Zuzana Walker, Gill Livingston

**Affiliations:** ^1^ Division of Psychiatry University College London London UK; ^2^ North London NHS Foundation Trust London UK; ^3^ Department of Statistical Science University College London London UK; ^4^ Research Department of Primary Care and Population Health University College London London UK; ^5^ Faculty of Medicine and Health Sciences University of Nottingham Nottingham UK; ^6^ Division of Psychology and Language Sciences University College London London UK; ^7^ North East London NHS Foundation Trust Rainham UK; ^8^ Tees Esk and Wear Valleys NHS Foundation Trust York UK; ^9^ Sir Jules Thorn Sleep and Circadian Neuroscience Institute, Nuffield Department of Clinical Neurosciences University of Oxford Oxford UK; ^10^ Centre for Dementia Studies Brighton and Sussex Medical School Brighton UK; ^11^ Essex Partnership University NHS Foundation Trust Essex UK

**Keywords:** clinical effectiveness, dementia, non‐pharmacological intervention, randomized controlled trial, sleep

## Abstract

**INTRODUCTION:**

Sleep disturbances are common and distressing for people with dementia and their family caregivers, with limited treatment options. The DREAMS START (Dementia RElAted Manual for Sleep; STrAtegies for RelaTives) multi‐component intervention for sleep disturbance in people at home with dementia is clinically and cost‐effective at 8 months. In this long‐term follow‐on study, we assessed 2‐year clinical effectiveness.

**METHODS:**

We recruited dyads of people with dementia and their family caregivers from community settings, for a two‐arm, multi‐center, single‐blind, parallel‐arm, superiority trial with the primary outcome Sleep Disorders Inventory (SDI). Analyses were intention to treat.

**RESULTS:**

We randomized 377 dyads, 189 to treatment‐as‐usual (TAU) and 188 to intervention; 177 dyads (46.9%) were followed up at 24 months. Two‐year adjusted mean SDI score was lower in the intervention arm than TAU (−5.40; 95% CI −9.14 to −1·67; *p* = 0·005).

**DISCUSSION:**

In this follow‐on study we demonstrate 2‐year improvement in sleep disruption for people with dementia. DREAMS START has potential for delivery at scale.

## Highlights

- We conducted a long‐term follow‐up of the DREAMS START (Dementia RElAted Manual for Sleep; STrAtegies for RelaTives) randomized controlled trials (RCT).
- We compared the DREAMS START intervention with usual care after 24 months.
- DREAMS START improved sleep in people with dementia at 2‐year follow‐up.
- DREAMS START did not improve family caregiver sleep at 2‐year follow‐up.

## 1 BACKGROUND

The 57 million people living with dementia worldwide is projected to increase to 153 million by 2050 and the cost of care is estimated to be 1 trillion United States Dollars.^1^ Dementia can affect sleep, resulting in impaired sleep initiation, reduced night‐time sleep, difficulty staying asleep, increased night‐time wandering, and excessive daytime sleepiness; these disturbances can be distressing and persistent for people with dementia and their families.^2^

In our meta‐analysis of sleep disturbance in people living at home with dementia, we found a pooled prevalence of 26% experiencing symptoms of sleep disturbance at any time, with 19% experiencing clinically significant sleep disturbance.^3^ Poor sleep may increase agitation and aggression^4^ and lead to reduced attention, motivation, and social engagement.^5^ Family caregivers find it difficult to cope with disturbed sleep,^6^, ^7^ which predicts family caregiver depressive symptoms, burden, and care home admission,^8^, ^9^ and is associated with increased resource use and health‐care costs.^10^

Sleep disturbance in dementia is related to neurodegeneration in brain structures involved in sleep and circadian rhythm regulation, including the suprachiasmatic nucleus.^11^ Additionally, over 90% of people with dementia have at least one other long‐term health condition and may experience pain, discomfort, or mood disturbances impacting sleep.^12^ Finding effective treatments with sustained effects is imperative for people living with dementia, their families and the services and communities supporting them.

Until recently there were no definitive randomized controlled trials (RCT) demonstrating sustained efficacy of pharmacological, non‐pharmacological, or light‐based treatment for sleep disturbance in dementia.^9^, ^13^ Relative stability in prevalence rates of sleep disturbance in dementia over the past two decades implies little progress in available treatments.^3^ Pharmacological interventions, such as antipsychotics and hypnotics have adverse effects^13^ including increased mortality in older adults,^14^ and there is a lack of evidence for the sustained efficacy and safety of existing medications.^13^, ^15^, ^16^, ^17^ A Cochrane review of non‐pharmacological interventions in dementia concluded that multi‐component, complex interventions have the strongest potential to improve sleep disturbance, with guidance recommending these as first‐line treatment^9^, ^15^

DREAMS START (Dementia RElAted Manual for Sleep; STrAtegies for RelaTives) is a feasible, acceptable, clinically effective, cost‐effective, and cost saving multi‐component intervention, codesigned to address the varied reasons for sleep disturbance.^18^, ^19^, ^20^, ^21^ The six session intervention incorporates psychoeducation, routine, light (natural and phototherapy through a light box), ways to manage distress behaviors at night, increased activity, exercise, relaxation, and caregiver's support and integrates actigraphic data from wrist‐worn devices to personalize strategies for people with dementia. It is manualized so the intervention can be delivered consistently, flexible so that it can be delivered online or in person and it is delivered by psychology graduates without specialist clinical training, so has potential for delivery at scale. Effectiveness was demonstrated immediately following intervention completion (4 months) and at 8‐month follow‐up. Family caregivers in the intervention arm also reported significantly improved sleep and reduced anxiety relative to the control arm and the intervention was cost‐effective after 8 months.^20^, ^21^ To our knowledge this was the first multi‐component non‐pharmacological intervention to demonstrate persistent effectiveness at 8 months; however, whether these effects are sustained in the longer‐term remains unclear.

This study aims to determine the long‐term (2 years from baseline) clinical effectiveness of DREAMS START for people with dementia and their family caregivers. Our primary objective was to determine whether DREAMS START continues to improve sleep disturbance in people with dementia living at home at 2‐year follow‐up compared to usual treatment.

## 2 METHODS

### 2.1 Study design and participants

**RESEARCH IN CONTEXT**

1. **Systematic review**: The authors reviewed the literature on non‐pharmacological interventions for sleep disturbance in dementia using established databases (e.g. MEDLINE and PsycINFO). Most published studies had small sample sizes and short follow‐up time periods. We found potential benefits of multicomponent interventions. No study demonstrated a sustained clinical effect on sleep in people with dementia.
2. **Interpretation**: Our findings show that the improvement in sleep for people with dementia demonstrated in the DREAMS START RCT after 8 months are sustained at long‐term follow‐up (24 months) relative to those who received usual care. Previously no such treatments have demonstrated such sustained impact.
3. **Future directions**: Multi component interventions like DREAMS START can lead to sustained improvement in sleep for people living at home with dementia. Future questions remain regarding best pathways for implementation at scale in different settings and for diverse populations.

DREAMS START is a two‐armed, multi‐center, parallel‐arm, superiority, pragmatic RCT with masked outcome assessment which recruited participant dyads of people living with dementia and their family caregivers from 12 English National Health Service (NHS) sites via community dementia services and from Join Dementia Research (JDR), a National Institute of Health and Care Research (NIHR) run free, secure online and telephone service for potential dementia research participants. The trial is registered ISRCTN (13072268) and was approved by London ‐ Camden & Kings Cross Ethics Committee (20/LO/0894) on August 21, 2020, and an amendment to conduct the 2‐year follow up was agreed on February 10, 2023. The published protocol can be accessed at https://bmjopen.bmj.com/content/14/2/e075273 ^22^ detailed protocol at https://www.isrctn.com/ISRCTN13072268; full methods are reported elsewhere.^21^

We recruited people: (i) with a documented diagnosis of any dementia type and severity; (ii) scoring ≥4 (clinically significant sleep disturbance) on any item on the Sleep Disorders Inventory (SDI), a valid and reliable tool for people with dementia which they or their family judged problematic; and (iii) who lived in their own home with a caregiver present at night. People with dementia were excluded if they had a known primary sleep disorder preceding dementia, were currently drinking heavily (Alcohol Use Disorders Identification Test ‐ Consumption (AUDIT C) Score ≥8), were unavailable for > 3 weeks during the trial, or were enrolled in another non‐pharmacological dementia RCT. We included family caregivers with capacity to give informed consent who supported their relative at least weekly practically or emotionally.

### 2.2 Randomization and masking

Participants were randomized 1:1 (intervention: treatment as usual [TAU]) at a participant (dyad) level, using random permuted blocks of sizes 2, 4, and 6 and stratified by site, using a Web‐based secure system (www.sealedenvelope.com) provided by the PRIMENT Clinical Trials Unit. We did not conduct further stratification as this was a pragmatic trial with a moderate sample size and additional stratification was not essential to ensure balance in characteristics between the groups. Researchers collecting outcome data were masked to allocation.

### 2.3 Procedures

On entering the RCT, participants (people with dementia and family caregivers) assessed as having capacity to consent to participate gave written or audio recorded informed consent. Assessments were informed by the Mental Capacity Act (2005), and all assessors had completed Good Clinical Practice training and had additional training in assessing mental capacity delivered by the trial manager and clinicians. When participants with dementia lacked capacity, family members acted as consultees. Mental capacity was reviewed by each assessor at each follow‐up point to account for changes in participants over time and in line with our research protocol. Data were collected from family caregivers at baseline, 4 months, 8 months, and 2 years. We obtained additional funding to conduct the longer‐term follow‐up study part way through the RCT and as such all participants were reconsented using the same procedures prior to 2‐year follow‐up data collection. All person with dementia‐related outcome measures were proxy reported by family caregivers in an effort to minimize burden for people with dementia and to ensure consistency of data. If family caregivers agreed, we audio‐recorded one randomly selected intervention session to assess intervention fidelity.

### 2.4 Intervention

**FIGURE 1:**
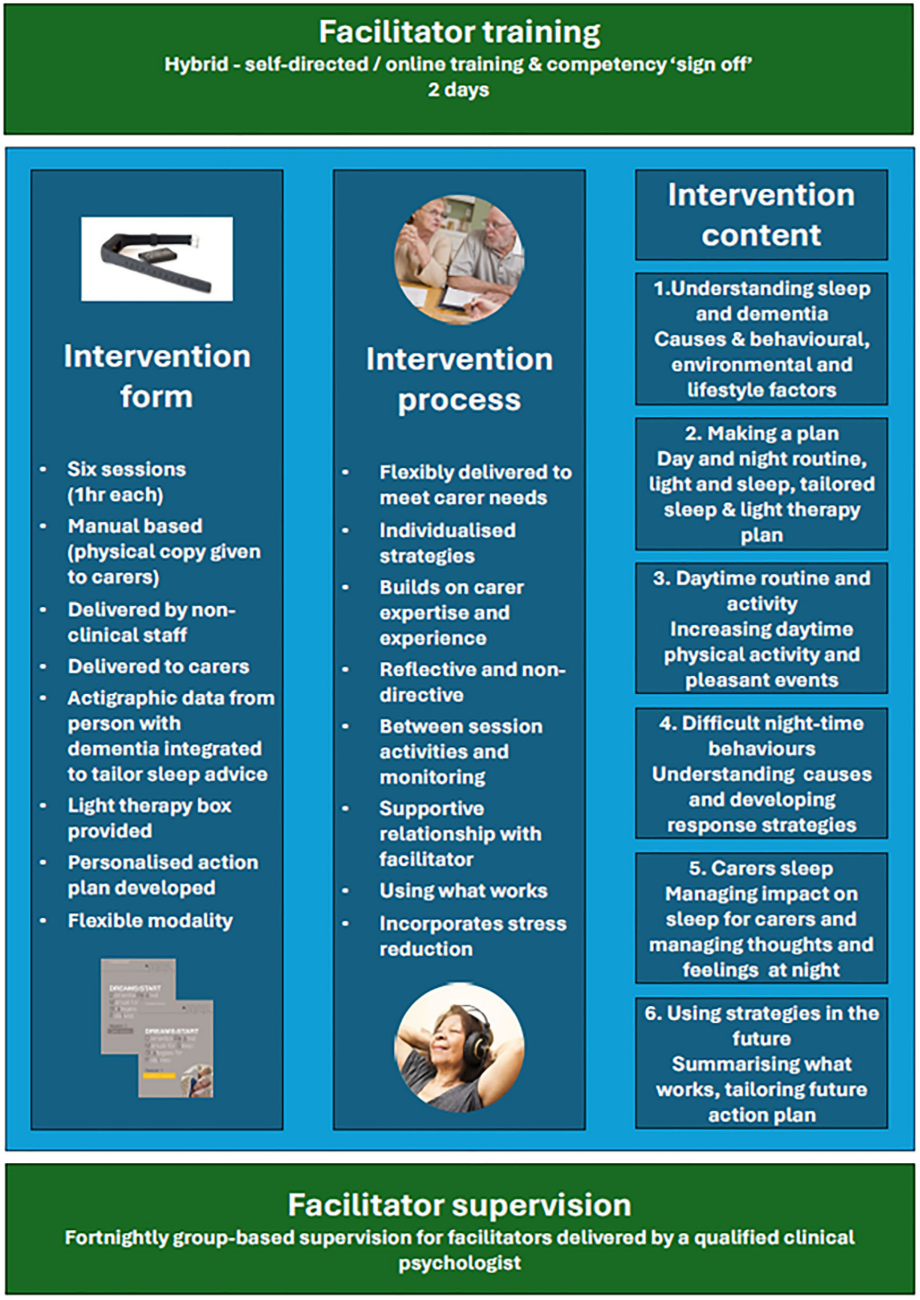
DREAMS START (Dementia RElAted Manual for Sleep; STrAtegies for RelaTives) intervention.

DREAMS START is a co‐produced, six‐session (1 hour/session) manualized, multi‐component intervention for caregivers of people with dementia to develop and implement personalized strategies for their relatives’ sleep via a tailored action plan. The intervention development, logic model, and underlying potential mechanisms are described in our earlier work. ^18^, ^23^ Sessions were delivered to caregivers weekly or fortnightly, in person, online via video call, or by telephone depending on preference and coronavirus disease 2019 (COVID‐19) restrictions (see Figure 1 for an overview of DREAMS START).

We trained and supervised non‐clinically qualified psychology graduates (facilitators) to deliver DREAMS START. Facilitators participated in a two‐day online training program delivered by our team. Training focused on dementia and sleep–wake regulation, empathic listening skills, behavior change, interpreting actigraphy, using supervision, and working collaboratively with family caregivers and people with dementia. Prior to delivery facilitators were signed off as ready to deliver the intervention by a research team clinician. Facilitators attended fortnightly group supervision with a team clinical psychologist with additional individual advice available. This process of “sign off” involved facilitators role playing the six intervention sessions fully with team clinicians P.R., G.L., or C.C., facilitators received live feedback in small groups and were asked to repeat until judged to be delivering to an adequate standard. If family caregivers agreed, we audio‐recorded one randomly selected intervention session to assess intervention fidelity. Fidelity rating was informed by the MRC framework for process evaluation^24^ and process factors were informed by our DREAMS START logic model, based on the Theoretical Domains Framework^25^ (described in published process evaluation^23^). We applied content checklists (see Appendix 1), and a mean fidelity score was produced by dividing the number of items delivered by the number of items that should be delivered. We pre‐defined 81%–100% as high, 51%–80% moderate, and <50% low fidelity. We also rated four intervention process factors which were: managing the concerns of the caregiver, keeping the caregiver engaged, keeping the caregiver focused on the manual and keeping to time, each rated from 1 not at all to 5 very much, and from this produced a mean score for the process items. Raters were familiar with the intervention and were trained to ensure consistency in rating fidelity with high inter‐rater reliability when each rater completed a rating also rated by a team clinician.

We had a usual care comparator rather than an active control condition. TAU within the NHS incorporates National Institute for Health and Care Excellence (NICE) guidelines for dementia consisting of assessment, diagnosis, symptomatic interventions, risk assessment and management, advice, and information.^15^ All participants received TAU which varies geographically and according to need and service configuration but might include medication for sleep. During our feasibility trial^19^ and our main RCT^21^, reported service use included a range of health and social care interventions which were primarily what would be expected as part of routine dementia care in NHS community services. There was a lack of specific sleep intervention offered other than use of medication. As we recruited from diverse settings in England, we believe this reflects what is typically offered in services.

### 2.5 Data collection

We collected demographic and illness characteristics at baseline. These were age, sex, ethnicity, and education of the person with dementia and caregiver, relationship of caregiver to recipient, dementia subtype diagnosed, and severity measured using the Clinical Dementia Rating (CDR).^26^

The primary outcome was sleep disturbance in people with dementia on the SDI 2 years after randomization. The same primary outcome had been assessed after 8 months for medium‐term effects.

The SDI is a family caregiver proxy‐measure, validated against clinical variables, measuring frequency (scale 0 [not present in the past 2 weeks] to 4 [once or more per day every night) and severity (scale 1 [mild] to 3 [marked]). Each item is calculated by multiplying frequency by severity (possible scores 0–12), with lower scores indicating better sleep.^27^ A decrease of ≥4 points is the suggested minimum clinically important difference (MCID).^28^ It is derived from the sleep domain of the Neuropsychiatric Inventory (NPI).^29^ Questions encompass: (i) difficulty falling asleep; (ii) getting up during the night; (iii) wandering, pacing, or getting involved in inappropriate activities at night; (iv) awakening the caregiver during the night; (v) awakening at night, dressing, and planning to go out, thinking that it is morning and time to start the day; (vi) awakening too early in the morning (earlier than is his/her habit); and (vii) sleeping excessively during the day. The SDI was collected at baseline, 4, 8, and 24 months.

Pre‐specified secondary outcomes were measured at baseline, 4, 8, and 24 months. A reduced set of outcomes was collected at 24 months to minimize burden on participants. For the person with dementia (all proxy‐rated) these were: DEMQOL‐Proxy, a valid and reliable 31‐item measure of dementia‐specific quality of life (scores range from 31 to 124, and lower scores indicate worse quality of life),^30^, ^31^ safety and tolerability assessment to record falls, dizziness, headaches, and gastrointestinal symptoms (appetite or bowel symptoms) and other possible side effects, whether mild, moderate, or severe; psychotropic medication (recorded via the Client Service Receipt Inventory [CSRI]^32^ and later classified as four types: anxiolytics and hypnotics, antipsychotics, antidepressants, adjuvant psychotropics, and melatonin); the occurrence and timing of death of the person living with dementia or date of any permanent move to a care home. For the family caregiver these were: Sleep Condition Indicator (SCI) an eight‐item scale to assess caregiver sleep disturbance (score range from 0–32, and are converted to a 10 point scale, higher scores indicate better sleep);^33^ Hospital Anxiety and Depression Scale (HADS), a 14‐item scale measuring anxiety and depression (anxiety and depression scores both range 0–21, higher scores indicate worse depression or anxiety);^34^ Zarit Burden Interview (ZBI) for caregiver burden is a 22‐item scale (scores range from 0 to 88, higher scores indicate more burden).^35^

### 2.6 Statistical analysis

The detailed statistical analysis plan (SAP) for the main trial, including sample size justification is pre‐registered at https://doi.org/10.1186/ISRCTN13072268 and the SAP for the 24‐month analyses presented here is available in Appendix 2.

In the primary analysis, the intervention effect at 24 months was estimated as a difference in mean SDI scores between intervention and control arms, calculated with a 95% confidence interval. This estimate was obtained from a three level, linear mixed effects multiple regression model using repeated measurements at 4, 8, and 24 months. The model included random effects to account for repeated outcomes and clustering by facilitator in the intervention arm, and fixed effects for treatment group, site, baseline SDI score, time point, and an interaction between treatment and time. We used similar models to estimate treatment effects for continuous secondary outcomes (DEMQOL‐Proxy, SCI, HADS, ZBI).

For psychotropic medications we summarized, by group, the frequency (%) of participants who took each type of medication (anxiolytics and hypnotics, antipsychotics, antidepressants, adjuvant psychotropics, and melatonin) during the 4‐month, 8‐month, and 24‐month follow‐up periods. Randomized groups were formally compared in terms of the proportions who had taken at least one type of medication (within those categories listed above) during the 24‐month follow‐up, using a difference in proportions and odds ratio with 95% confidence intervals. Odds ratios were estimated using a mixed effects logistic regression model with fixed and random components mirroring those used in the primary analysis. A similar mixed effects binomial generalized linear model did not converge, so estimates of the difference in proportions at 24 months were obtained from a simplified model that did not use repeated measurements.

The time to care home admission (for a permanent move) or death was calculated relative to baseline and summarized by randomized group using Kaplan–Meier plots for each event type and the composite (death or care home admission). Those lost to follow‐up before admission/death were censored at their last follow‐up point. To formally compare groups, the time to admission/death were analyzed using a parametric shared frailty model, assuming a Weibull survival model, allowing for facilitator clustering in the intervention arm and adjusting for study site as a fixed effect. We also fitted a competing risks model, adjusted for study site, to obtain separate effect estimates (sub‐hazard ratios) for death and care‐home transition.

We used an intention to treat approach and included all available data, assuming any missing/unavailable values were missing at random (MAR). For our primary outcome, we carried out sensitivity analyses to consider the impact of missing data on our results. We refitted our main model after multiple imputation of missing values. The imputation model included repeated measurements of SDI, site, and any other variables possibly related to missingness (age, gender, ethnicity, marital status, level of education, living situation and accommodation of participants with dementia and caregiver, caregiver's relationship to participants with dementia, age at dementia diagnosis, and dementia diagnosis). The imputations were performed by study arm and estimates combined using Rubin's rules. A pattern mixture approach was also used to considered missing not at random (MNAR) scenarios in particular reflecting that those who move to a care home are likely to have worse sleep (see Appendix 2 p.8). In addition (taking a “while alive” approach) we carried out a set of analyses using only outcome data at 24 months (not the repeated measurements) (see Appendix 2 p.8). All analyses were conducted using Stata version 18.0

### 2.7 Role of the funding source

The funder of the study had no role in the study design, data collection, analysis, interpretation, manuscript drafting, or decision to submit to publication.

## 3 RESULTS

### 3.1 Participant flow

**FIGURE 2:**
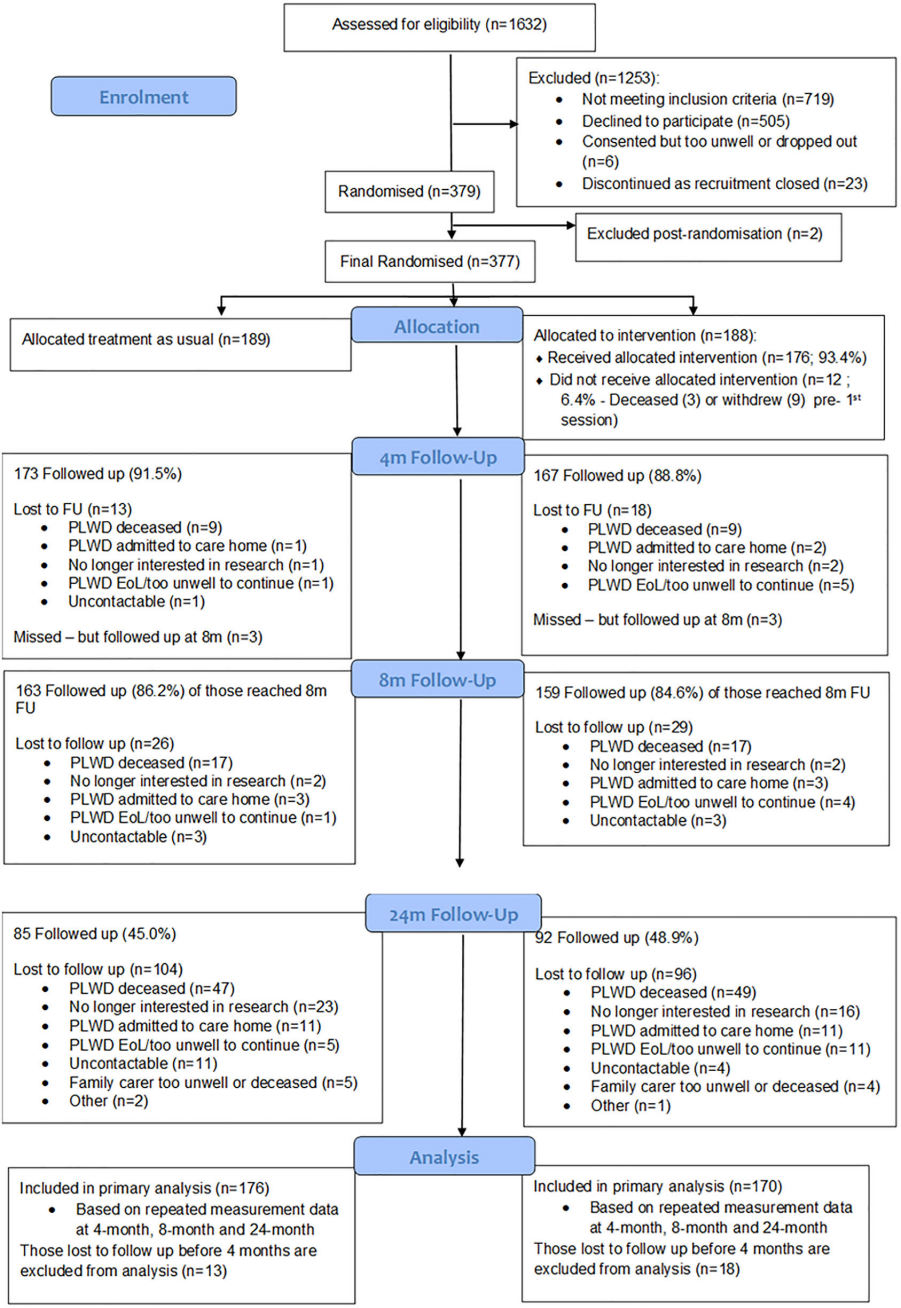
Participant flow through trial. EoL, end of life; FU, follow‐up; PLwD, person living with dementia; RCT, randomized controlled trial.

Between February 24, 2021, and March 5, 2023, we randomized 377 dyads; of those 189 were allocated to TAU and 188 to the intervention. Of the 377 dyads randomized, 177 dyads (46.9%) were followed up at 24 months; of these 92 were allocated to intervention and 85 were allocated to TAU. Of those lost to follow‐up at 24 months, 96 (25.5%) participants with dementia had died and 22 (5.8%) had been admitted to a care home. The CONSORT diagram (see Figure 2) shows participant flow through the study.

Baseline demographic characteristics of participants with dementia and caregivers are shown in Tables 1 and 2 and were similar across arms. Of those followed up at 24 months demographic characteristics were similar across arms although there were more men living with dementia and more female and spousal caregivers in the TAU arm (see Appendix 3 Tables 1 and 2 for characteristics of those followed up at 24 months compared to total randomized).

**TABLE 1:** Baseline characteristics of people with dementia by trial arm.

Parameter	Treatment as usual (*n* = 189)	Intervention (*n* = 188)
Age (years): mean (SD)	79.1 (9.3)	79.7 (8.7)
Sex		
Male	85 (45.0%)	86 (45.7%)
Female	104 (55.0%)	102 (54.3%)
Marital status		
Single	6 (3.2%)	5 (2.7%)
Widowed	48 (25.4%)	48 (25.5%)
Married/civil partnership	117 (61.9%)	123 (65.4%)
Cohabiting	5 (2.6%)	3 (1.6%)
Separated	4 (2.1%)	2 (1.1%)
Divorced	9 (4.8%)	7 (3.7%)
Level of education		
Postgraduate degree	22 (11.6%)	18 (9.6%)
Undergraduate Degree	20 (10.6%)	25 (13.3%)
A level (or equivalent)	19 (10.1%)	16 (8.5%)
HNC/HND (or equivalent)	11 (5.8%)	14 (7.4%)
NVQ (or equivalent)	9 (4.8%)	11 (5.9%)
GCSE (or equivalent)	16 (8.5%)	22 (11.7%)
School leaving certificate	33 (17.5%)	39 (20.7%)
No formal qualifications	52 (27.5%)	36 (19.1%)
Other	7 (3.7%)	7 (3.7%)
Ethnicity		
White	142 (75.1%)	140 (74.5%)
Mixed	1 (0.5%)	2 (1.1%)
Asian	24 (12.7%)	23 (12.2%)
Black	14 (7.4%)	18 (9.6%)
Arab	4 (2.1%)	1 (0.5%)
Other	4 (2.1%)	4 (2.1%)
Dementia diagnosis		
Alzheimer's disease	102 (54.0%)	104 (55.3%)
Frontotemporal dementia	7 (3.7%)	7 (3.7%)
Vascular dementia	34 (18.0%)	30 (16.0%)
Lewy body dementia	13 (6.9%)	16 (8.5%)
Posterior cortical atrophy dementia	1 (0.5%)	1 (0.5%)
Progressive supranuclear palsy	0 (0.0%)	1 (0.5%)
Parkinson's disease	3 (1.6%)	3 (1.6%)
Mixed dementia	22 (11.6%)	19 (10.1%)
Alcohol related	1 (0.5%)	0 (0.0%)
Semantic dementia	0 (0.0%)	1 (0.5%)
Unable to specify	6 (3.2%)	6 (3.2%)
Living situation		
Lives alone/ someone present at night	9 (4.8%)	8 (4.3%)
Lives with children	57 (30.2%)	43 (22.9%)
Lives with partner/spouse	111 (58.7%)	120 (63.8%)
Lives with flat/housemates	1 (0.5%)	1 (0.5%)
Other	11 (5.8%)	16 (8.5%)
Type of accommodation		
Council rented	16 (8.5%)	19 (10.1%)
Owner‐occupied	149 (78.8%)	142 (75.5%)
Housing association rented	11 (5.8%)	9 (4.8%)
Private rented	10 (5.3%)	16 (8.5%)
Other	3 (1.6%)	2 (1.1%)

*Note*: Data are *n* (%) or mean (SD).

Abbreviations: GCSE, general certificate of secondary education; HNC, higher national certificate; HND, higher national diploma; NVQ, national vocational qualification.

**TABLE 2:** Baseline characteristics of caregivers by trial arm.

Parameter	Treatment as usual (*n* = 189)	Intervention (*n* = 188)
Age (years): mean (SD)	63.5 (12.9)	64.6 (13.7)
Sex		
Male	52 (27.5%)	66 (35.1%)
Female	137 (72.5%)	122 (64.9%)
Marital status		
Single	32 (16.9%)	36 (19.1%)
Widowed	8 (4.2%)	3 (1.6%)
Married/civil partnership	125 (66.1%)	136 (72.3%)
Cohabiting	11 (5.8%)	4 (2.1%)
Separated	4 (2.1%)	2 (1.1%)
Divorced	9 (4.8%)	7 (3.7%)
Relationship of caregiver to care recipient		
Spouse/partner	96 (50.8%)	105 (55.9%)
Friend	1 (0.5%)	1 (0.5%)
Child	82 (43.4%)	75 (39.9%)
Other	10 (5.3%)	7 (3.7%)
Currently living with care recipient		
Yes	160 (84.7%)	163 (86.7%)
No	29 (15.3%)	25 (13.3%)
Level of education		
Postgraduate degree	38 (20.1%)	37 (19.7%)
Undergraduate degree	42 (22.2%)	39 (20.7%)
A level (or equivalent)	25 (13.2%)	28 (14.9%)
HNC/HND (or equivalent)	18 (9.5%)	12 (6.4%)
NVQ (or equivalent)	10 (5.3%)	12 (6.4%)
GCSE (or equivalent)	33 (17.5%)	27 (14.4%)
School leaving certificate	12 (6.3%)	16 (8.5%)
No formal qualifications	11 (5.8%)	10 (5.3%)
Other	0 (0.0%)	7 (3.7%)
Ethnicity		
White	144 (76.2%)	139 (73.9%)
Mixed	1 (0.5%)	5 (2.7%)
Asian	25 (13.2%)	23 (12.2%)
Black	14 (7.4%)	16 (8.5%)
Arab	1 (0.5%)	1 (0.5%)
Other	4 (2.1%)	4 (2.1%)

*Note*: Data are *n* (%) or mean (SD).

Abbreviations: GCSE, General Certificate of Secondary Education; HNC, Higher National Certificate; HND, Higher National Diploma; NVQ, National Vocational Qualification.

### 3.2 Intervention fidelity and adherence

In total, 149/180 (82.8%) of surviving intervention participants adhered to the intervention, receiving ≥ four of six intervention sessions, 142 received all six sessions, and eight participants died before completing four sessions. The intervention was delivered by 49 facilitators who delivered to between one and 15 participants each. Delivery methods varied with 77 (43·8%) participants receiving the intervention in person, 31 (17·6%) by video call, 28 (15·9%) by telephone, and 40 (22.7%) having mixed delivery formats. The mean intervention fidelity score was 95·4% (SD 0·08). For all process measures assessed, the median score was 5/5 (interquartile range [IQR] 5 to 5).

### 3.3 Primary outcome

**FIGURE 3 alz71274-fig-0003:**
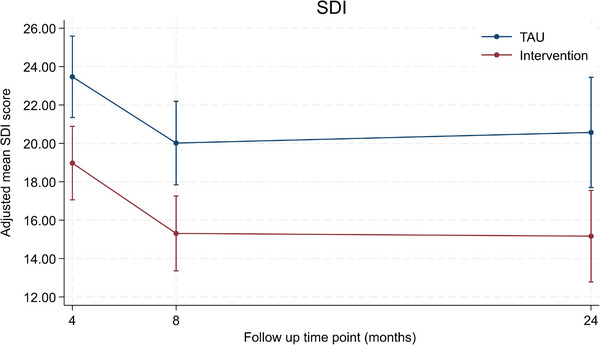
Adjusted mean SDI scores (circles) with 95% confidence intervals (vertical bars) at 4, 8, and 24 months by randomized group, TAU, the adjusted mean and confidence intervals are estimated from the primary analysis model. SDI, Sleep Disorders Inventory [higher scores indicate greater sleep disturbance]; TAU, treatment as usual.

Average SDI scores at 24 months were 14.4 (SD 11.4, *n* = 92) in the intervention arm and 19.2 (SD 14.7, *n* = 84) for TAU (see Table 3 & Figure 3). We included 346 participants with dementia who had at least one post‐randomization SDI score (at 4, 8, or 24 months) in our primary mixed effects model. From this model, the adjusted mean difference was −5.40 points (95% CI −9.14 to −1.67, *p* = 0.005, *n* = 346). Sensitivity analyses for the primary outcome including multiple imputation and using a pattern mixture approach to consider MNAR scenarios gave similar findings to the primary model (see Appendix 3 pp.5–9).

**TABLE 3:** Main analysis of primary and secondary outcomes for people living with dementia.

Parameter	Treatment as usual		Intervention		Adjusted mean difference (95% CI; *n*)
	*n*	Mean (SD)	*n*	Mean (SD)	
SDI					
Baseline	189	33.25 (17.70)	188	31.86 (16.43)	…
4 months	173	23.64 (18.66)	167	18.99 (14.98)	−4.49 (−7.35 to −1.63); 346
8 months	163	20.34 (16.67)	159	15.16 (12.77)	−4.71 (−7.63 to −1.78); 346
24 months	84	19.2 (14.7)	92	14.4 (11.4)	−5.40 (−9.14 to −1.67); 346
DEMQOL‐Proxy					
Baseline	187	90.22 (14.70)	187	92.06 (14.59)	…
4 months	128	92.88 (14.53)	138	96.54 (13.23)	1.53 (−0.98 to 4.04); 278
8 months	112	96.20 (11.21)	124	96.76 (12.33)	−1.38 (−4.01 to 1.25); 278
24 months	63	99.38 (10.56)	80	98.72 (11.18)	−0.06 (−3.25 to 3.13); 278

*Note*: Higher scores indicate worse outcome for SDI. Lower scores indicate worse outcome for DEMQOL‐Proxy.

Abbreviations: CI, confidence interval; DEMQOL‐Proxy, Dementia Quality of Life; SD, standard deviation; SDI, Sleep Disorders Inventory.

### 3.4 Comparison of secondary outcomes

There were no statistically significant differences between arms in any of our secondary outcome scales or medication use at 24 months for people with dementia (see Table 3 and Appendix 3 pp.10–12). The previously significant improvement in caregiver sleep and anxiety at 8 months was not sustained at 2 years (see Table 4). There were no notable safety events (see Appendix 3 p.12).

**TABLE 4 alz71274-tbl-0004:** Main analyses of secondary outcomes for caregivers

Parameter	Treatment as usual		Intervention		Adjusted mean difference (95% CI; *n*)
	*n*	Mean (SD)	*n*	Mean (SD)	
SCI score					
Baseline	189	4.37 (2.48)	188	4.35 (2.60)	
4 months	129	4.91 (2.67)	141	5.03 (2.77)	0.09 (−0.39 to 0.58); 300
8 months	124	4.92 (2.67)	135	5.50 (2.69)	0.60 (0.11 to 1.10); 300
24 months	73	5.90 (2.67)	91	5.65 (2.80)	0.14 (−0.46 to 0.75); 300
HADS Anxiety score					
Baseline	189	8.21 (4.40)	188	8.51 (4.60)	
4 months	127	7.06 (4.48)	137	7.05 (4.48)	−0.44 (−1.25 to 0.38); 279
8 months	111	6.75 (4.26)	120	6.29 (4.28)	−0.85 (−1.71 to 0.01); 279
24 months	61	5.57 (3.49)	75	6.11 (3.82)	−0.45 (−1.51 to 0.62); 279
HADS Depression score					
Baseline	189	6.32 (4.31)	188	6.47 (4.38)	
4 months	127	6.08 (3.99)	137	6.18 (4.37)	−0.13 (−0.89 to 0.63); 279
8 months	111	6.17 (4.26)	120	6.08 (4.30)	−0.30 (−1.10 to 0.50); 279
24 months	61	4.67 (3.54)	75	5.08 (4.27)	−0.68 (−1.67 to 0.30); 279
HADS Total score					
Baseline	189	14.53 (7.63)	188	14.97 (7.84)	
4 months	127	13.14 (7.42)	137	13.23 (8.04)	−0.64 (−1.97 to 0.69); 279
8 months	111	12.92 (7.48)	120	12.38 (7.59)	−1.21 (−2.61 to 0.19); 279
24 months	61	10.25 (5.97)	75	11.19 (7.15)	−1.19 (−2.93 to 0.54); 279
Zarit score					
Baseline	187	36.24 (16.43)	186	35.08 (18.44)	
4 months	124	35.47 (15.04)	138	32.72 (16.34)	−1.50 (−4.17 to 1.17); 277
8 months	109	33.80 (14.76)	120	31.83 (15.85)	−0.12 (−2.92 to 2.68); 277
24 months	61	28.90 (14.59)	75	30.33 (16.57)	1.43 (−1.99 to 4.85); 277

*Note*: Lower score indicates worse outcome for SCI. Higher score indicates worse outcome for HADS‐Depression and HADS‐Anxiety and Zarit.

Abbreviations: CI, confidence interval; HADS,  Hospital Anxiety and Depression Scale; SCI,  Sleep Conditions Indicator; SD, standard deviation.

### 3.5 Time to care‐home admission and death

**FIGURE 4 alz71274-fig-0004:**
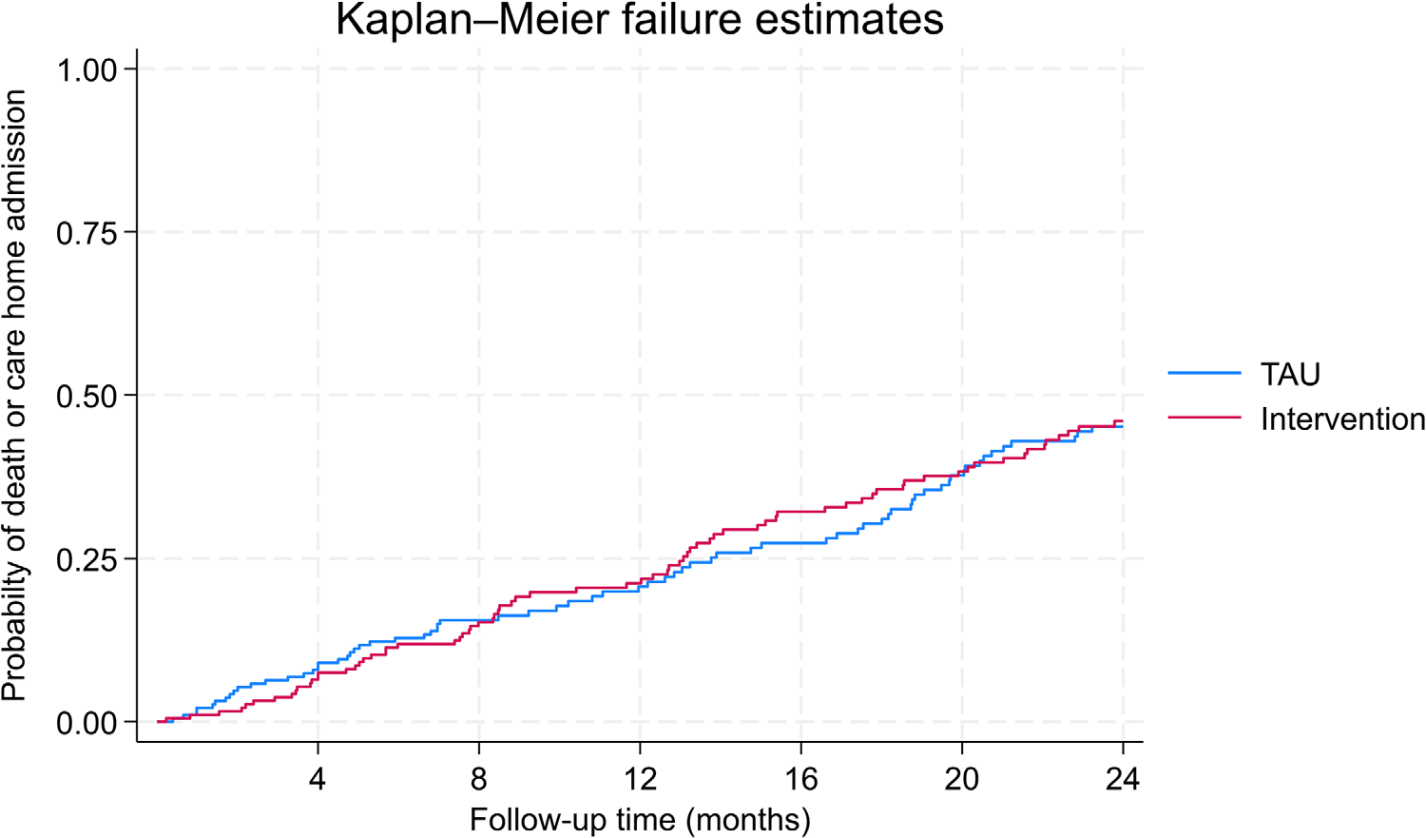
Kaplan–Meier plot for probability of death or care‐home admission.

At 24 months, 37 (23.3%) intervention and 34 (20.9%) TAU participants with dementia were known to have moved to a care home, of whom 11 (intervention) and eight (TAU) had subsequently died within 24 months. Overall, 49 (30.8%) intervention and 47 (28.8%) TAU participants with dementia were known to have died. Figure 4 shows the Kaplan–Meier plot for the composite outcome of dying or moving to a care home. Results from adjusted analyses show no evidence of a difference between groups; the adjusted hazard ratio for dying or moving to a care home was 1.02 (95% CI 0.70,1.50) for the intervention compared to TAU. Sub‐hazard ratios for death and care‐home admissions were 1.01 (95% CI 0.61,1.67) and 1.12 (95% CI 0.68,1.86), respectively (see Appendix 3 pp13–15).

## 4 DISCUSSION

The DREAMS START manualized multi‐component intervention resulted in improved sleep for people living at home with dementia 2 years after recruitment and around 20 months after intervention delivery had finished when compared to usual treatment. This is the first non‐drug sleep intervention to demonstrate long‐term clinical effectiveness; the change in SDI scores exceeds the MCID, suggesting that the improvement is important to people with dementia, their families, and the services supporting them. With high levels of attrition after 24 months, we must be cautious in interpreting our findings; however, sensitivity analyses on our primary outcome, including a range of prespecified scenarios, produced similar estimates of treatment effectiveness, providing reassurance that the findings are robust and unlikely to be driven by the missing values. Our DREAMS START process evaluation highlighted how developing knowledge and skills in managing sleep in dementia, adapting strategies and strengthening routines as circumstances changed, led to observable improvements for caregivers after 8 months, although these were not sustained at 24 months. Tailored, long‐term action plans may have reinforced caregivers’ sense of competence and ability to manage and respond, encouraging them to continue to use helpful behavioral, environmental, and psychological strategies so that they embedded and sustained changes well beyond the end of the intervention period, resulting in positive long‐term effects on their relative with dementia's sleep.^23^

The[Fig alz71274-fig-0003], [Table alz71274-tbl-0004] improvement in caregiver sleep and anxiety observed at 8 months following DREAMS START intervention was not sustained at 2 years, and we found no evidence of differences in any of our other secondary outcomes.^21^ Interestingly, across both trial arms, we saw a trend for improvement in caregiver sleep, burden, and mental health outcomes and person with dementia quality of life from baseline to 24 months despite the expected course of illness. This may reflect participants being recruited during the COVID‐19 pandemic era when caregivers are known to have experienced worse mental health and higher burden, and people with dementia worse quality of life due to social isolation and poorer access to services and support.^36^, ^37^, ^38^ We also found that the DREAMS START intervention did not change the likelihood of people with dementia moving to care homes, although more intensive, nonpharmacological interventions specifically focused on maintaining people with dementia at home, have previously demonstrated a delay to care home transition.^39^

As noted above, there are a lack of fully powered evaluations of interventions for sleep in dementia with long‐term follow‐up data which makes our study unique.^9^, ^13^ We were able to include data on 92% of participants in our primary analysis. Our sample was socially and ethnically diverse, which gives our findings good ecological validity. We did have high levels of attrition, largely due to participant death, illness, and care‐home transition. The DREAMS START intervention was delivered to a high standard of fidelity across different modalities, which indicates the potential for consistent delivery in diverse settings. Limitations include the possibility of response bias as we relied on family caregiver proxy and self‐report outcome measures, and we were unable to mask participants and facilitators to group allocation. This is common in psychological and behavioral trials^40^ and we took steps to mitigate against the impact of researchers becoming unblinded to allocation status by the person facilitating the intervention being different from the researcher conducting follow‐up assessments and reminding participants not to mention the intervention to the assessor and to hide materials or equipment. Unblinding was minimal, assessors were unblinded at five of the 4‐month and two of the 8‐month follow‐up assessments, and a different researcher completed subsequent assessments. At the 24‐month follow‐up, assessors were unblinded twice. Additionally, we did not include actigraphy or any other direct sleep or activity measure as an outcome for the trial. This decision was based on our earlier feasibility RCT, feedback from family caregivers on what were meaningful and important outcomes for them,^19^ and inconsistencies between questionnaire and actigraphy data reported in other studies.^41^, ^42^

Previously, no[Fig alz71274-fig-0004] non‐pharmacological intervention for sleep disturbance in dementia has been ready for scaling up and widespread intervention, despite promise from multi‐component interventions.^9^ DREAMS START is the only non‐pharmacological evidence‐based intervention package incorporating all aspects of the UK NICE recommendations.^15^ We have developed DREAMS START as a mixture of product and procedure: a structured, manualized, transferable package incorporating practice guidelines and a lay summary and video animation for commissioners and training materials (the animation can be viewed here. We have also made recommendations on using the intervention with those with multiple long‐term conditions^43^ and for UK South Asian communities^44^ and are working on adapting the intervention specifically for those with dementia with Lewy bodies and Parkinson's disease, although they were included in the trial. We are currently researching optimal pathways to implementation to maximize potential reach and impact across diverse settings.^45^ As post‐diagnostic support interventions are typically delivered within NHS memory services or third‐sector community settings and similar interventions such as START^46^ tend to be delivered in these settings, DREAMS START is likely to fit best in these services. Memory services often have assistant psychologists and support workers who would be well placed to facilitate the intervention with supervision from qualified clinical psychologists. Additionally, there is potential to adapt DREAMS START for care‐home settings where sleep problems are very common and distressing for residents with dementia and we have begun this work.

The long‐term cost‐effectiveness of the intervention is currently being investigated and will be published at a later date. Additionally, having accurate, acceptable, valid, and reliable measurement of direct sleep and movement remains important, and future testing of entirely passive and non‐intrusive technology for people living at home with dementia is needed.^47^, ^48^ This would also further elucidate the underlying mechanisms of action for DREAMS START, for example, circadian rest–activity rhythms and changes in pattern of light exposure, sleep–wake timing, and consolidation and reduction in napping. Being able to quantitatively explore these mechanisms would enable us to better understand which aspects of the intervention work for whom, and in what ways, which has the potential to lead to further personalization and targeting of the intervention.

In conclusion, DREAMS START is clinically effective in the long‐term reducing sleep disturbance in people living at home with dementia at 2‐year follow‐up, although the impact on caregiver sleep which we saw after 8 months was not sustained. Sleep disturbance in dementia is common, distressing, and disruptive for people with dementia and their families, whilst safe, effective treatments are currently not widely available. Our study presents a viable and safe treatment option, and as such, DREAMS START is a strong candidate for being made widely available in health and care services.

## CONFLICTS OF INTEREST STATEMENT

P.R. declares grants from NIHR Academy, NIHR PGfAR with no COI with current work. G.L., P.R., and J.B. are supported by University College London Hospitals’ National Institute for Health Research (NIHR) Biomedical Research Centre, and G.L. is also supported by North Thames NIHR Applied Research Collaboration and as an NIHR Senior Investigator and has grants from NIHR PGfAR, Alzheimer's Association, Norwegian Research Council and Wellcome with no COI with current work. S.B. declares grants from NIHR, CIHR, ESRC, HEE, ESPRC, Alzheimer's Society, and the Alzheimer's Association with no COI with current work. C.E. declares grants from NIHR‐HTA, NIHR‐EME, NIHR‐BRC, Wellcome Trust with no COI with current work. S.D.K. declares grants from NIHR Oxford Health BRC (NIHR203316), NIHR‐HTA (12/87/61 and 16/84/01), NIHR‐EME (NIHR131789), NIHR PGfAR (NIHR203667), Wellcome Trust (226784/Z/22/Z and 227093/Z/23/Z).) and MRC (MR/Z506540/1). MR declares a grant from NIHR ARC KSS and ARUK with no COI with current work. G.C. is part‐funded by ESRC/NIHR/Alzheimer's Society with no COI with current work. Outside the submitted work S.K. declares non‐financial support from Big Health Ltd. in the form of no cost access to the digital sleep improvement programme, Sleepio, for use in clinical research. Outside the submitted work CE declares stock/stock options from Big Health Limited developers of Sleepio. Outside the submitted work SB declares personal fees and non‐financial support from Lilly, personal fees from Boehringer‐Ingelheim, personal fees from Axovant, personal fees from Lundbeck, personal fees from Nutricia and honoraria from the Hamad Medical Service for lectures and talks. S.B. is a trustee of the Alzheimer's Society and NED at Nottingham University Hospital NHS Trust. Outside the submitted work, M.R. declares Honorarium for presentation on Lewy body dementias for GE. C.E. is deputy editor of the Journal of Sleep Research and on the editorial board of Sleep Medicine reviews. All other authors declare no competing interests. Author disclosures are available in the Supporting Information.

## CONSENT STATEMENT

All participants provided informed consent unless they did not have capacity to do so in which case their relevant representative provided informed consent on their behalf for participation.

## Supporting information



Supporting information

Supporting information

Supporting information

Supporting information

## Data Availability

The datasets generated during and/or analyzed during the current study are available upon request from PRIMENT Clinical Trials Unit (CTU) Data Management Group on priment@ucl.ac.uk in collaboration with members of DREAMS START Trial Team. Any request for data must come through to PRIMENT CTU in the first instance and where the request is reasonable, anonymised datasets, stored on the publicly available UCL Research Data Repository https://rdr.ucl.ac.uk/ will be shared.
