# Supplementary material for: The DREAMS START intervention for sleep in dementia: Long‐term follow‐up of a randomized controlled trial
Source: Alzheimers Dement. 2026 Mar 11;22(3):e71274. doi: 10.1002/alz.71274 (PMC13093547; doi:10.1002/alz.71274)
Supplement: Supplementary file 1 — Supporting information [file ALZ-22-e71274-s003.docx]

Check List (DREAMS START) session 1

Facilitator Name:……………………………………………………..

Assessor Name: ………………………………………………………

Participant ID:………………………………………………………….

Delivered to: …………………………………………………………..

Mode of delivery:…………………………………………………….

Length of session:……………………………………………………

Date: ……………………………………………………………………….

| Introduction | | Y/N | |
| --- | --- | --- | --- |
| All information covered (Inc. key point) or **if not,** an explanation is given for why not covered. | |  | |
| What to expect | |  | |
| All information covered | |  | |
| Outline that carer will put strategies in place during each week | |  | |
| Sleep and dementia | |  | |
| All information covered | |  | |
| Discuss carer quotes | |  | |
| Facilitate discussion and create list of sleep problems person with dementia maybe experiencing | |  | |
| What is sleep? | |  | |
| Facilitate discussion of talking point: What do you notice happening to you and your relative when you don’t get a good night’s sleep? | |  | |
| All information covered | |  | |
| Outline diagram | |  | |
| How does sleep work? | |  | |
| All information covered (Inc. Key Point) | |  | |
| Outline diagrams and checked that they have understood. If not, clarify. | |  | |
| Why is sleep important? | |  | |
| All information covered | |  | |
| Facilitate discussion of talking point: Did your relative have problems sleeping before the diagnosis, if so, what helped then? | |  | |
| What causes sleep problems in dementia? | |  | |
| All information covered | |  | |
| Facilitate discussion of talking point: working through example given and discussing potential causes for own relatives sleep problems. | |  | |
| Making changes to improve sleep | |  | |
| All information covered | |  | |
| Discuss lifestyle and bedroom environment factors impact on carer and relative | |  | |
| Facilitate lifestyle/ bedroom environment changes for the relative | |  | |
| Make a plan for changes and encourage carer to write plan down on record form | |  | |
| The impact of sleep problems on you and your relative | |  | |
| All information covered (Inc. Key point) | |  | |
| Discuss carer quotes | |  | |
| Facilitate discussion around sleep problems and which problems are deemed most distressing for both carer and relative | |  | |
| Refer to telecare and additional leaflet if relevant | |  | |
| Managing the stress of sleep problems | |  | |
| All information covered (Inc. Key point) | |  | |
| Facilitate discussion around carer stress/ emotions | |  | |
| The signal breath | |  | |
| All information covered | |  | |
| Teach signal breath | |  | |
| Rate stress before and after | |  | |
| Putting it into practice | |  | |
| All information covered | |  | |
| Summarise the session | |  | |
| Remind carer to practice stress reduction technique | |  | |
| Discuss sleep diary with carer | |  | |
| Discuss Planning record | |  | |
| Troubleshoot around ‘putting it into practice’ between session tasks | |  | |
| Process factors | | Facilitator (1 not at all – 5 very much) | |
| Keeping the session to time | |  | |
| Keeping the carer focussed on the manual | |  | |
| Keeping the carer engaged in the session | |  | |
| Managing concerns (relevant to intervention) of the carer | |  | |
