# Supplementary material for: The DREAMS START intervention for sleep in dementia: Long‐term follow‐up of a randomized controlled trial
Source: Alzheimers Dement. 2026 Mar 11;22(3):e71274. doi: 10.1002/alz.71274 (PMC13093547; doi:10.1002/alz.71274)
Supplement: Supplementary file 2 — Supporting information [file ALZ-22-e71274-s001.pdf]

Priment™ Clinical Trials Unit

UCL

DREAMS TRIAL EXTENSION: STATISTICAL ANALYSIS PLAN (SAP)

Full title of trial: A parallel multi-centre randomised controlled trial to determine the clinical and cost-effectiveness of DREAMS START (Dementia RElAted Manual for Sleep; STrAtegies for RelaTives) for people living with dementia and their carers

Version 1.0

Started: 28th January 2025

Prepared by: Julie Barber & Mariam Adeleke

Based on protocol version 5 (27th Feb 2024)

Authors	Title	Signature	Date
Dr Julie Barber	Co-ap statistician		25th March 2025
Dr Mariam Adeleke	Trial statistician		10/04/2025
Reviewers	Title	Signature	
Dr Penny Rapaport	Chief Investigator		03/04/2025
Prof Gill Livingston	Chief Investigator		03/04/2025
Approver	Title	Signature	Date
Dr Penny Rapaport	Chief-Investigator		03/04/2025

Version History Log

Version	Date	Details of change
0.1	13th March 2025	
1.0	25th March 2025	Update after review by listed reviewers

1. TRIAL SUMMARY

This summary of the DREAMS START trial is based on the study protocol (version 5). For full detailed information see the study protocol.

1.1 TRIAL DESIGN

Individually randomised controlled trial with process evaluation in people living with dementia and their family carers (Dyads). Dyads were randomised in a 1:1 ratio to the DREAMS START intervention or treatment-as-usual with randomisation blocked and stratified by site. Participants were followed up at 4, 8 and 24 months after randomisation. Primary outcome was SDI score at 8 months.

1.2 OBJECTIVES OF EXTENSION STUDY

Primary objective: To determine whether the DREAMS START intervention improves sleep disturbance in people living with dementia at home after 2 years compared to treatment-as-usual and to inform implementation discussions with trusts and policy makers.

Secondary objectives: To determine impacts of the intervention at 2 years in terms of

1. Whether it increases people with dementia's quality of life.
2. Whether it is cost-effective
3. Whether it leads to a delay in time to care home transition.
4. The role of psychotropic medication and melatonin in any change.
5. Whether it improves family carers' sleep and decreases their affective symptoms and burden.

1.3 OUTCOMES RELEVANT TO EXTENSION STUDY

1.4.1 PRIMARY OUTCOME

The primary outcome is the extent of sleep disturbance the person with dementia is experiencing after 24 months as measured using the sleep disorders inventory (SDI). The SDI has seven sleep sub-questions: difficulty falling asleep; getting up during the night (not scored as positive if someone gets up once or twice per night to pass urine and quickly falls back to sleep); wandering, pacing or getting involved in inappropriate activities at night; awakening the carer during the night; awakening at night, dressing, and planning to go out, thinking that it is morning and time to start the day; awakening too early in the morning (earlier than is his/her habit); and sleeping excessively during the day. Each item is rated according to frequency (scale 0 (Not present in the last two weeks) – 4 (once or more per day (every night)) and severity (scale 1 (mild) -3 (marked)) of sleep-disturbed behaviours and, when multiplied possible item scores range from 0-12. Data will be treated as continuous. This instrument is collected at baseline, 4, 8 month and 24 months follow up.

1.4.2 SECONDARY OUTCOMES

The following secondary outcome measures will be considered in extension analyses. All were recorded at baseline, 4, 8 and 24 month follow ups.

Person living with dementia (all proxy measures):

1. DEMQOL-Proxy is a 31 item interviewer-administered questionnaire measuring quality of life in people with dementia. Items are scores as A lot = 1; Quite a bit = 2; A little = 3; Not at all = 4. In calculating a total score, positive items are reversed so that for all items a higher score means better quality of life. (Items: 1, 4, 6, 8, 11 and 32 need reversing). Total score is the sum across 31 items, giving a range for the total score from 31 to 124. Higher scores indicate better QL. (As recommended, where less than 50% of the items are missing, these items will be imputed using the within person mean from all available items. Total scores will not be calculated if there are insufficient (<50%) items).
2. Medication- psychotropic medication to delineate the role of rescue medication and any effect of intervention on prescribing. This data will be collected as part of the CSRI.
3. Side effects - Using a Safety, and Tolerability Assessment to record the occurrence of falls, dizziness, headaches and gastrointestinal symptoms (appetite or bowel symptoms) and other side effects and whether these were mild, moderate or severe. This will allow us to assess potential harms.
4. Modified Client Service Receipt Inventory (CSRI) (Beecham and Knapp, 1992) a proxy questionnaire asking about health and social care service use information in the past 4 months for the patient (including care home admission, extra patient care during therapy). The analysis of this outcome measure will be described in the health economic plan.
5. EQ-5D 5 level (EQ-5D-5L)[49] proxy is a generic measure of health related quality of life. Carer proxy responses will be used to calculate QALYs and incremental cost per QALY gained. The analysis of this outcome measure will be described in the health economic plan.

Family carer:

1. Sleep Condition Indicator (SCI) is an eight item scale to assess sleep disturbance. It characterises sleep both dimensionally and against insomnia disorder criteria. Each item scored 0 to 4. Total SCI score is calculated as the sum of all scores giving a range 0 to 32 (a higher score meaning better sleep).
2. The hospital anxiety and depression scale (HADS) is a 14 item measured used to detect the states of depression and anxiety using two separate subscales. Carers are asked to rate, on a 4-point scale (0-3), different aspects of their mood. 7 items related to anxiety and 7 to depression. By summing the items, 3 scores can be calculated: HADS-Depression (sum of the depression items) & HADS-Anxiety (sum of anxiety items) which range from 0 (low severity) – 21 (high severity) and HADS-Total score (sum of HADS-D and HADS-A) ranging from 0 to 42.
3. Zarit Burden Interview (ZBI) measures the impact that care giving has on the carer. A 22-item self-report questionnaire asks different aspects of how people feel taking care of another person on a scale of 0-4. This results in an accumulated score ranging from 0 (no burden) – 88 (severe burden).
4. Modified Client Service Receipt Inventory (CSRI) a questionnaire asking about health and social care service use information in the past 4 months. This will incorporate the Valuation of

Informal Care Questionnaire (iVICQ) a measure of carer time and activity and the Brief Work Productivity and Activity Impairment (WPAI) a measure of productivity loss. The analysis of this outcome measure will be described in the health economic plan.

5. EQ-5D 5 level (EQ-5D-5L) is a generic measure of health related quality of life. The analysis of this outcome measure will be described in the health economic plan.

In addition, information has been recorded about the occurrence and timing of death of the person living with dementia or date and timing of any permanent move to a care home.

2. DETAILED ANALYSIS PLAN

This analysis plan gives detail of the main quantitative statistical analyses of effectiveness and safety outcomes for the DREAMS trial extension study (up to 24 months). These analyses should be viewed as secondary since the primary analysis for the main trial has been completed and published. Plans for analysis of 24 month health economic outcomes and qualitative information will be covered separately. The analyses described in this document do not preclude the undertaking of further ad-hoc analyses, although the results of any such further analyses would be interpreted carefully. Furthermore, the SAP does not prevent the adaption of any part of the trial analysis, should situations arise in which such adaptation is necessary. Any such adaptation will be fully justified and transparent.

2.1 TIMING OF ANALYSIS

The extension trial analyses will take place once this SAP is formally signed off and the database that includes all data up to 24 months has been locked.

2.2 DATA CHECKING

Before analysis and database lock, basic checks will be performed on the quality of the data, focusing on identifying:

- Missing data
- Data outside expected range
- Other inconsistencies between variables e.g. in the dates the questionnaires were completed

If any inconsistencies are found, the corresponding values will be double checked with the researchers and corrected if necessary in the source data. This checking process and subsequent changes will be documented.

2.3 STATISTICAL PRINCIPLES

Analyses will be planned and conducted according to the principles of GCP, the research governance framework, and ICH topic E9 'Statistical Principles for Clinical Trials' and following the SOPs of the PRIMENT clinical trials unit. Results will be reported following Consort guidance.

2.3.1 Analysis population & Primary outcome Estimands

The main analysis will aim to estimate the effect of the intervention at 24 months compared with standard care, regardless of the post-randomisation (intercurrent) events listed below for the case where the person living with dementia is alive at 24 months. The analysis will exclude those randomised in error as previously agreed by the trial steering committee/ DMEC.

The primary estimand attributes are defined below:

**Estimand Aspect**		
Population	Dyads meeting the eligibility criteria	
Treatment condition	Up to 6 sessions of intervention + standard care compared with standard care alone, regardless of compliance with treatment, treatment discontinuation or use of rescue medications	
Endpoint	SDI at 24 months	
Summary measure	Mean difference	
<u>*Handling of intercurrent events*</u>		
Study treatment discontinuation/non-compliance	Treatment policy	
Use of rescue / other medications or interventions	Treatment policy	
Death of PLWD	A: Hypothetical strategy (treatment effect estimated assuming no deaths within 24 months)	B : While alive strategy (treatment effect estimated amongst those alive after 24 months)
Death of Carer	Treatment policy	
Care home admission	Treatment policy	
Hospitalisation of carer or PLWD	Treatment policy	

Where possible, outcome data was collected after each of the listed intercurrent events. Where data is missing for reasons other than death, we will assume that the participant's outcome after their intercurrent event will be similar to that of all other participants (MAR). We will expect the death rate over a 2 year period in this population to be fairly high, although similar between randomised groups. To address death we will calculate estimands based on 2 different strategies: The primary approach (A) will take a hypothetical strategy, mirroring the previous DREAMS analyses of data up to 8 months. Here data unavailable due to death will be treated as missing (MAR) and the treatment effect estimated under the hypothetical scenario that people do not die within 24 months. A secondary approach (B) will use a 'while alive strategy' where analysis approaches <u>do not</u> impute (explicitly or implicitly) the outcomes for those who have died. Extent, reasons and characteristics of those without data will be examined. Sensitivity analyses will be used to consider the impact of the primary approach for handling unavailable/missing data on the results.

2.3.2 Confidence intervals and P-values

Confidence intervals will be presented at the 95% level and, along with P-values, will be 2 sided for all analyses.

2.3.3 Statistical software

Analyses will be conducted by Mariam Adeleke and Julie Barber using Stata version 18 (or above) (StataCorp 2023). R may be used for multi-level multiple imputation.

2.4 Description of study sample

2.4.1 Consort diagram

A consort diagram will be constructed to describe the flow of subjects through the trial (http://www.consort-statement.org/). The diagram will detail the number of subjects: approached and assessed for eligibility; eligible; agreeing to enter the study (with reasons for refusal); receiving the intervention (with reason for not receiving this); followed-up at each stage, including numbers who entered the 24 month extension study; numbers withdrawn/died (with reasons) & the numbers analysed for the primary outcome at 24 months.

2.4.2 External validity

Baseline characteristics of the people living with dementia and the carers who were followed up at 24 months will be summarised by treatment group to gauge whether a balance in characteristics between the randomised groups remains. The results will be presented as means with standard deviations for continuous, symmetric variables, medians and interquartile ranges for continuous, skewed variables, and frequencies and percentages for categorical variables. No significance testing will be used.

2.4.4 Protocol deviations

Timing of 4, 8 & 24 month data collection relative to baseline will be reported by randomised group, including the proportion with assessments outside of the pre-specified window (+/- 4 weeks around the 4,8, 24 month date relative to baseline). Any other protocol deviations will be summarised.

2.4.5 Attrition and missing data

Loss to follow-up is expected over 24 months, particularly due to death. Reasons for 24 month outcome data being unavailable will be described and frequency (%) of subjects with missing data, by reason and timepoint will be provided for each randomised group (and for each outcome).

2.5 Analysis of primary outcome

2.5.1 Summary information

For each randomised group we will summarise the primary outcome (SDI at 24 months) using means with standard deviations and medians with interquartile ranges. We will also graphically examine the distribution of the scores. We will examine repeated measurements of the SDI outcome at 4, 8 and 24 months by treatment group using summary statistics and profile plots.

2.5.2 Main analysis (estimand A)

The effect of the intervention will be described using the difference in mean 24-month SDI scores (between intervention and control groups) calculated with a 95% confidence interval and P-value.

This estimate will be obtained from a 3 level, linear mixed effects multiple regression model which allows analysis of repeated outcome measurements at 4, 8 and 24 months and for clustering by facilitator in the intervention arm. The model will include a treatment group indicator, time indicator, indicators for the interaction between treatment and time, baseline SDI score and indicators for site as fixed effects. We will fit the heteroscedastic mixed effects model described by Candlish *et. al*, 2018. Control group participants will be treated as clusters of size 1. Participants in the intervention group where no intervention was received and a facilitator was not assigned will also be treated as clusters of 1. We will use adjusted degrees of freedom (kenward-roger, Stata option *dfmethod(kroger)*) and restricted maximum likelihood (Stata option *REML*) for estimation, as recommended.

Example STATA code for this model:

mixed SDI SDI$_0$ i.randgrp##i.time i.site, || clusterid: randgrp, nocons ||participantid:,
residual(independent , by(randgrp)) dfmethod(kroger) reml

The intra-cluster correlation coefficient (with 95% confidence interval) will be calculated to describe facilitator clustering.

Analysis will include all those with available data. This model assumes all missing/unavailable data are missing at random.

In the event of the model not fitting
If the model with heteroscedastic residuals does not converge, we will first fit a model with homoscedastic residuals. If convergence issues remain, we will first fit the model without site and if necessary, also exclude facilitator clustering.

In the event of non-normal residuals
Model assumptions will be checked. SDI scores are expected to be slightly positively skewed, however with adjustment for baseline SDI included, the model residuals are likely to be approximately normally distributed. If, however, residuals are found to be severely non normal, the main model will be refitted after suitable transformation of the SDI score (e.g. a log transformation).

2.5.3 SENSITIVITY ANALYSES FOR MISSING OUTCOME (ESTIMAND A)

We will carry out a sensitivity analysis for the primary outcome using multiple imputation assuming that missing/unavailable data are MAR. The imputation model will include repeated measurements of the outcome of interest, socio-demographic baseline data and any other variables possibly related to missingness and the outcome. The imputations will be performed by study arm, allowing for clustering. The primary analysis model will then be re-fitted using the imputed data. We will use the number of imputations that is around the proportion of missingness (e.g. 20 imputed sets for 20% missing data) and combine the results using Rubin's rules (R may be used here to enable multi-level imputation).

We will use pattern mixture models for sensitivity analysis under MNAR. These will involve modifying the MAR imputed data (as created for the sensitivity analysis 2) above) to reflect agreed MNAR scenarios (adding a specified factor d to imputed values), fitting the primary model and combining estimates using Rubin's rules (Cro *et al,* 2020).

This method will be used to impute missing SDI values with the following conditions:

a) If the participant has missing outcome data because they were admitted to a care home, then we will add *d1* to the MAR imputed SDI scores.
b) If the participant has missing outcome data due to end of life or death we will add *d2* to MAR imputed values.
c) If the participant has missing outcome data for any other reason, data will remain as previously imputed.

Given disturbed sleep is often a predictor of entry into care, we might expect worse sleep for those that move to a care home. For *d1* we will consider increases of 25%, 50% and 75% of the absolute change of the SDI score observed over 24 months (from baseline value) for all participants.

It is difficult to predict how death/end of life will impact on sleep so *d2* will take a broader range of values considering increases and decreases of 25%, 50% and 75% of the absolute change of the SDI score over 24 months based on all participants.

2.5.4 ANALYSES – ESTIMAND B

The secondary estimand of interest is defined similarly to estimand A but with death handled using a 'While alive' strategy (treatment effect estimated amongst those alive at 24 months).

The estimate of interest will be obtained from a 2 level, linear (heteroscedastic) mixed effects multiple regression model of the 24 month SDI score allowing for clustering by facilitator in the intervention arm (similarly to the primary model). The model will include a treatment group indicator, baseline SDI score and indicators for site as fixed effects. This model will assume missing outcome for those who didn't die are MAR. Those who died within 24 months will not be considered in this analysis.

As a secondary approach to estimate the same estimand, we may explore using a joint model for survival (time to death) and longitudinal SDI scores over 24 months. We will model SDI follow up scores using a linear mixed model with fixed effects for the treatment group, baseline SDI, site and follow up time indicators, and treatment-by-time interactions. We will model the survival process assuming a

Weibull survival distribution, with fixed effects for treatment group and study site. We will present only estimates and confidence intervals from the longitudinal mixed component of the model (to minimise complexity models will not allow for facilitator clustering, which has previously been shown to be minimal). The joint models will be obtained using the *stjm* command in STATA (or may use R).

Missing data sensitivity analyses similar to those described in 2.5.3 will also be conducted for this estimand. Both sensitivity analysis approaches will use the same imputation sets as previously generated but any imputations made after death will be removed before fitting of the analysis model.

2.6 Analysis of secondary outcomes (excluding those for health economic analyses)

2.6.1 Continuous secondary outcomes

The following secondary outcomes produce continuous scores:

Person with dementia: DEMQOL-Proxy

Family carer: SCI, HADS - anxiety, HADS - depression, HADS – total, ZBI

Analyses carried out for these scores will be similar to those described for the primary outcome (section 2.5.1, 2.5.2 & 2.5.4). Main analyses will focus on outcome at 24 months.

Sensitivity analyses for missing outcome

Similar sensitivity analyses as planned for the primary outcome will be considered for secondary outcomes with concerning amounts of missing data. (section 2.5.3)

2.6.2 Binary secondary outcome: Psychotropic medication

The frequency (%) of participants in each randomised group who have taken at least one type of medication during the 4-month, 8-month & 24-month follow-up period will be calculated. The frequency (%) of each type of medication (anxiolytics and hypnotics, antipsychotics, antidepressants, adjuvant psychotropics, and melatonin) will also be summarised.

Randomised groups will be compared in terms of the proportions who have taken at least one type of medication (within those categories listed above), obtaining an estimate of the difference in proportions and odds ratio with 95% confidence intervals at 24 months.

The difference in proportions will be estimated using a mixed effects binomial generalised linear model with identity link (Stata command *gllamm* with options *link(id) family(binomial)*). The model will include as fixed effects, indicators for treatment group, follow-up time, an interaction between treatment and time, site and a covariate for baseline psychotropic medication. Facilitator and participant identifier will be represented as random effects.

Odds ratios will be estimated using a similar mixed effects logistic regression model.

Estimates will be obtained for 4, 8 & 24 month follow up points.

2.6.3 'SURVIVAL' TYPE SECONDARY OUTCOMES

We will summarise the proportion of participants known to have transitioned from home (i.e. proportion who had either moved into a care home for a permanent move or who had died), the proportion who had moved to a care home (for a permanent move) and the proportion who had died at each follow-up time point by randomised group.

The time to care home admission (for a permanent move) or death will be calculated relative to baseline and summarised by randomised group using Kaplan-Meier plots (for the 2 events separately, and for the composite). Those lost to follow up before admission/death will be treated as censored. To formally compare groups the time to admission/death will be analysed using a parametric shared frailty model, allowing for facilitator clustering in the intervention arm and adjusting for study site as a fixed effect.

Supportive analysis

The time to care home admission (for a permanent move) and death will be compared separately between randomised groups using a competing risks model, adjusting for study site. The competing risks model will estimate the marginal hazard rates for care home admission and death. The model will be fitted using 'stcrreg' command in stata. Facilitator clustering was found to be minimal in the primary analysis and will be ignored for these complex models.

2.6.4. SIDE EFFECTS

Side effects - which may not be side effects but part of illnesses- (occurrence of falls, dizziness, headaches and gastrointestinal symptoms (appetite or bowel symptoms) and other side effects) and whether these were mild, moderate or severe will be summarised by randomised group using frequency (%).

2.7 REFERENCES

StataCorp. 2023. *Stata Statistical Software: Release 18*. College Station, TX: StataCorp LLC.

Candlish *et al.* BMC Medical Research Methodology (2018). 18:105 https://doi.org/10.1186/s12874-018-0559-x

Cro *et al,* Statistics in Medicine (2020). 39:21 https://doi.org/10.1002/sim.8569
