# Supplementary material for: The DREAMS START intervention for sleep in dementia: Long‐term follow‐up of a randomized controlled trial
Source: Alzheimers Dement. 2026 Mar 11;22(3):e71274. doi: 10.1002/alz.71274 (PMC13093547; doi:10.1002/alz.71274)
Supplement: Supplementary file 3 — Supporting information [file ALZ-22-e71274-s004.docx]

Appendix 3 DREAMS 24 month additional analyses.

**Prepared by: Dr Mariam Adeleke and Prof Julie Barber**

**Date: 18 September 2025**

## Comparison of randomised participants and those that were follow-up at 24-month

Baseline characteristics of all randomised participants are summarised alongside those for the subgroup who were followed-up at 24-months. The participants followed up at 24-months appear to be a good representation of participants that were randomised.

Table 1: Baseline characteristics of PLWD that were randomised and the subgroup who were followed up at 24-months. Values are number (%) unless stated otherwise.

|  | Randomised (n=377) | Followed up at 24m  (n=177) |
| --- | --- | --- |
| Site |  |  |
| 01 | 48 (12.7%) | 19 (10.7%) |
| 02 | 13 (3.4%) | 4 (2.3%) |
| 03 | 53 (14.1%) | 24 (13.6%) |
| 04 | 64 (17.0%) | 28 (15.8%) |
| 05 | 51 (13.5%) | 26 (14.7%) |
| 06 | 27 (7.2%) | 16 (9.0%) |
| 07 | 52 (13.8%) | 23 (13.0%) |
| 08 | 35 (9.3%) | 17 (9.6%) |
| 09 | 18 (4.8%) | 7 (4.0%) |
| 10 | 5 (1.3%) | 4 (2.3%) |
| 11 | 3 (0.8%) | 3 (1.7%) |
| 12 | 8 (2.1%) | 6 (3.4%) |
|  |  |  |
| Age (Years): Mean (SD) | 79.4 (9.0) | 77.9 (8.6) |
|  |  |  |
| Sex |  |  |
| Male | 171 (45.4%) | 75 (42.4%) |
| Female | 206 (54.6%) | 102 (57.6%) |
|  |  |  |
| Marital Status |  |  |
| Single | 11 (2.9%) | 5 (2.8%) |
| Widowed | 96 (25.5%) | 42 (23.7%) |
| Married/Civil partnership | 240 (63.7%) | 115 (65.0%) |
| Cohabiting | 8 (2.1%) | 3 (1.7%) |
| Separated | 6 (1.6%) | 1 (0.6%) |
| Divorced | 16 (4.2%) | 11 (6.2%) |
|  |  |  |
| Level of education |  |  |
| Postgraduate degree | 40 (10.6%) | 13 (7.3%) |
| Undergraduate Degree | 45 (11.9%) | 32 (18.1%) |
| A level (or equivalent) | 35 (9.3%) | 15 (8.5%) |
| HNC/HND (or equivalent) | 25 (6.6%) | 9 (5.1%) |
| NVQ (or equivalent) | 20 (5.3%) | 12 (6.8%) |
| GCSE (or equivalent) | 38 (10.1%) | 26 (14.7%) |
| School leaving certificate | 72 (19.1%) | 31 (17.5%) |
| No formal qualifications | 88 (23.3%) | 31 (17.5%) |
| Other | 14 (3.7%) | 8 (4.5%) |
|  |  |  |
| Ethnicity |  |  |
| White | 282 (74.8%) | 131 (74.0%) |
| Mixed | 3 (0.8%) | 2 (1.1%) |
| Asian | 47 (12.5%) | 22 (12.4%) |
| Black | 32 (8.5%) | 15 (8.5%) |
| Arab | 5 (1.3%) | 3 (1.7%) |
| Other | 8 (2.1%) | 4 (2.3%) |
|  |  |  |
| Dementia diagnosis |  |  |
| Alzheimer’s Disease | 206 (54.6%) | 98 (55.4%) |
| Frontotemporal dementia | 14 (3.7%) | 8 (4.5%) |
| Vascular dementia | 64 (17.0%) | 31 (17.5%) |
| Lewy body dementia | 29 (7.7%) | 12 (6.8%) |
| Posterior cortical atrophy dementia | 2 (0.5%) | 0 (0%) |
| Progressive supranuclear palsy | 1 (0.3%) | 1 (0.6%) |
| Parkinson's Disease | 6 (1.6%) | 5 (2.8%) |
| Mixed Dementia | 41 (10.9%) | 17 (9.6%) |
| Alcohol related | 1 (0.3%) | 1 (0.6%) |
| Semantic dementia | 1 (0.3%) | 1 (0.6%) |
| Unable to specify | 12 (3.2%) | 3 (1.7%) |
|  |  |  |
| Date of dementia diagnosis known? |  |  |
| Yes | 376 (99.7%) | 177 (100%) |
| No | 1 (0.3%) | 0 (0%) |
|  | 2.6 (2.7) | 2.4 (2.5) |
| Time (years) since dementia diagnosis (mean (SD)) |  |  |
| Age at diagnosis (mean (SD)) | 76.8 (9.1) | 75.5 (8.7) |
|  |  |  |
| Living situation |  |  |
| Lives alone with someone present at night | 17 (4.5%) | 7 (4.0%) |
| Lives with children | 100 (26.5%) | 41 (23.2%) |
| Lives with partner/spouse | 231 (61.3%) | 113 (63.8%) |
| Lives with flat/housemates | 2 (0.5%) | 1 (0.6%) |
| Other | 27 (7.2%) | 15 (8.5%) |
|  |  |  |
| Type of accommodation |  |  |
| Council rented | 35 (9.3%) | 17 (9.6%) |
| Owner-occupied | 291 (77.2%) | 137 (77.4%) |
| Housing association rented | 20 (5.3%) | 7 (4.0%) |
| Private rented | 26 (6.9%) | 14 (7.9%) |
| Other | 5 (1.3%) | 2 (1.1%) |

Table 2: Baseline characteristics of carers that were randomised and those that were followed up at 24-month. Values are number (%) unless stated otherwise.

|  | Randomised (n=377) | Followed up at 24m (n=177) |
| --- | --- | --- |
| Age (years): Mean (SD)^1^ | 64.1 (13.3) | 64.0 (12.4) |
|  |  |  |
| Sex |  |  |
| Male | 118 (31.3%) | 58 (32.8%) |
| Female | 259 (68.7%) | 119 (67.2%) |
|  |  |  |
| Marital Status |  |  |
| Single | 68 (18.0%) | 31 (17.5%) |
| Widowed | 11 (2.9%) | 2 (1.1%) |
| Married/Civil partnership | 261 (69.2%) | 128 (72.3%) |
| Cohabiting | 15 (4.0%) | 5 (2.8%) |
| Separated | 6 (1.6%) | 2 (1.1%) |
| Divorced | 16 (4.2%) | 9 (5.1%) |
|  |  |  |
| Relationship of carer to care recipient |  |  |
| Spouse/partner | 201 (53.3%) | 102 (57.6%) |
| Friend | 2 (0.5%) | 0 (0%) |
| Child | 157 (41.6%) | 68 (38.4%) |
| Other | 17 (4.5%) | 7 (4.0%) |
|  |  |  |
| Currently living with care recipient? |  |  |
| Yes | 323 (85.7%) | 155 (87.6%) |
| No | 54 (14.3%) | 22 (12.4%) |
| Level of education |  |  |
| Postgraduate degree | 75 (19.9%) | 42 (23.7%) |
| Undergraduate Degree | 81 (21.5%) | 40 (22.6%) |
| A level (or equivalent) | 53 (14.1%) | 27 (15.3%) |
| HNC/HND (or equivalent) | 30 (8.0%) | 13 (7.3%) |
| NVQ (or equivalent) | 22 (5.8%) | 9 (5.1%) |
| GCSE (or equivalent) | 60 (15.9%) | 25 (14.1%) |
| School leaving certificate | 28 (7.4%) | 10 (5.6%) |
| No formal qualifications | 21 (5.6%) | 7 (4.0%) |
| Other | 7 (1.9%) | 4 (2.3%) |
|  |  |  |
| Ethnicity |  |  |
| White | 283 (75.1%) | 130 (73.4%) |
| Mixed | 6 (1.6%) | 5 (2.8%) |
| Asian | 48 (12.7%) | 21 (11.9%) |
| Black | 30 (8.0%) | 15 (8.5%) |
| Arab | 2 (0.5%) | 1 (0.6%) |
| Other | 8 (2.1%) | 5 (2.8%) |
|  |  |  |
| Living situation |  |  |
| Lives alone | 7 (1.9%) | 3 (1.7%) |
| Lives with parent(s) | 91 (24.1%) | 38 (21.5%) |
| Lives with partner/spouse | 246 (65.3%) | 120 (67.8%) |
| Lives with children | 7 (1.9%) | 5 (2.8%) |
| Lives with flat/housemates | 2 (0.5%) | 1 (0.6%) |
| Other | 24 (6.4%) | 10 (5.6%) |
|  |  |  |
| Type of accommodation |  |  |
| Council rented | 36 (9.5%) | 16 (9.0%) |
| Owner-occupied | 295 (78.2%) | 139 (78.5%) |
| Housing association rented | 16 (4.2%) | 6 (3.4%) |
| Private rented | 27 (7.2%) | 15 (8.5%) |
| Other | 3 (0.8%) | 1 (0.6%) |

## Sensitivity analyses for missing outcome (estimand A)

37 participants are missing primary outcome at 4-months, 55 at 8-months and 201 at 24-months. Reasons for missingness at 24-months are given below

Table 3: Reason for missing 24-month SDI data

| Reason for missing 24-month data | Treatment as usual (n = 105) | Intervention (n = 96) |
| --- | --- | --- |
| SDI score |  |  |
| Death | 47 | 49 |
| Withdrew – End of life | 5 | 11 |
| Withdrew - Admitted to care home | 11 | 11 |
| Withdrew – other reasons | 41 | 25 |
| Outcome not measured | 1 | 0 |

Multiple imputation was carried out under the MAR assumption. The imputation model included baseline SDI score, site, and demographic data of PLWD and carers (age, gender, ethnicity, marital status, level of education, living situation, accommodation, carer’s relationship to PLWD, age of dementia diagnosis and dementia diagnosis). Two-level multiple imputation was carried out to account for the repeated measures at 4-month, 8-month and 24-month. We did not account for facilitator clustering because the model with facilitator did not converge. The imputation was carried out using *jomo* package in *R*. 60 imputed data sets were saved.

The main analysis model was fitted for each imputed data set and the estimates were combined using Rubin’s rules. This was done using *mi estimate* function in Stata, which does not support the Kenward-Roger method for adjusted degrees of freedom.

Table 4: Results of multiple imputation analysis of the primary outcome

|  | Adjusted mean difference (95% CI) |
| --- | --- |
|  |  |
| SDI |  |
| 4 months | -4.43 (-7.28 to -1.58) |
| 8 months | -4.62 (-7.56 to -1.68) |
| 24 months | -5.10 (-8.69 to -1.52) |

*Pattern mixture models under MNAR assumption*

The imputed data sets in the above are used here, the imputed SDI scores are modified depending on the reason for missing data:

1. If the participant has missing outcome data because they were admitted to a care home, then we will add *d1* to the MAR imputed SDI scores.
2. If the participant has missing outcome data due to end of life or death we will add *d2* to MAR imputed values.
3. If the participant has missing outcome data for any other reason, data will remain as previously imputed.

Given disturbed sleep is often a predictor of entry into care, we might expect worse sleep for those that move to a care home. For *d1* we will consider increases of 25%, 50% and 75% of the absolute change of the SDI score observed over 8 months (from baseline value) for all participants.

It is difficult to predict how death/end of life will impact on sleep so *d2* will take a broader range of values considering increases and decreases of 25%, 50% and 75% of the absolute change of the SDI score over 8 months based on all participants.

Table 5: Reasons for missingness at 4 months and 8 months

| Reason for missingness | Treatment as usual | Intervention |
| --- | --- | --- |
| Death/ End of life |  |  |
| 4 months | 10 | 14 |
| 8 months | 18 | 21 |
| 24 months | 52 | 60 |
| Admitted to care home |  |  |
| 4 months | 1 | 2 |
| 8 months | 3 | 3 |
| 24 months | 11 | 11 |

The 3-level models did not converge for these specifications, so a two-level model that accounted for repeated measures at 4-month, 8-month and 24-month was used. This model is also consistent with the imputation model.

Table 6: Results of pattern mixture models under MNAR assumption for varying values of *d1* and *d2*.

|  | Adjusted mean difference (95% CI) |
| --- | --- |
| SDI – *d1 = +25%; d2 = +25%* |  |
| 4 months | -4.17 (-7.15 to -1.19) |
| 8 months | -4.40 (-7.46 to -1.34) |
| 24 months | -4.58 (-8.23 to -0.93) |
| SDI – *d1 = +25%; d2 = +50%* |  |
| 4 months | -4.11 (-7.18 to -1.03) |
| 8 months | -4.34 (-7.49 to -1.18) |
| 24 months | -4.39 (-8.11 to -0.67) |
| SDI – *d1 = +25%; d2 = +75%* |  |
| 4 months | -4.04 (-7.22 to -0.86) |
| 8 months | -4.27 (-7.52 to -1.01) |
| 24 months | -4.20 (-8.00 to -0.39) |
| SDI – *d1 = +25%; d2 = -25%* |  |
| 4 months | -4.30 (-7.15 to -1.45) |
| 8 months | -4.53 (-7.46 to -1.60) |
| 24 months | -4.94 (-8.47 to -1.41) |
| SDI – *d1 = +25%; d2 = -50%* |  |
| 4 months | -4.35 (-7.16 to -1.54) |
| 8 months | -4.59 (-7.48 to -1.70) |
| 24 months | -5.12 (-8.60 to -1.63) |
| SDI – *d1 = +25%; d2 = -75%* |  |
| 4 months | -4.40 (-7.20 to -1.61) |
| 8 months | -4.64 (-7.50 to -1.78) |
| 24 months | -5.28 (-8.72 to -1.84) |
| SDI – *d1 = +50%; d2 = +25%* |  |
| 4 months | -4.16 (-7.15 to -1.16) |
| 8 months | -4.40 (-7.47 to -1.33) |
| 24 months | -4.57 (-8.23 to -0.92) |
| SDI – *d1 = +50%; d2 = +50%* |  |
| 4 months | -4.09 (-7.17 to -1.01) |
| 8 months | -4.33 (-7.49 to -1.17) |
| 24 months | -4.39 (-8.11 to -0.66) |
| SDI – *d1 = +50%; d2 = +75%* |  |
| 4 months | -4.02 (-7.21 to -0.83) |
| 8 months | -4.26 (-7.53 to -1.00) |
| 24 months | -4.19 (-8.00 to -0.38) |
| SDI – *d1 = +50%; d2 = -25%* |  |
| 4 months | -4.28 (-7.15 to -1.41) |
| 8 months | -4.53 (-7.47 to -1.58) |
| 24 months | -4.94 (-8.48 to -1.40) |
| SDI – *d1 = +50%; d2 = -50%* |  |
| 4 months | -4.33 (-7.16 to -1.50) |
| 8 months | -4.58 (-7.49 to -1.68) |
| 24 months | -5.11 (-8.60 to -1.61) |
| SDI – *d1 = +50%; d2 = -75%* |  |
| 4 months | -4.38 (-7.20 to -1.57) |
| 8 months | -4.63 (-7.51 to -1.76) |
| 24 months | -5.27 (-8.72 to -1.82) |
| SDI – *d1 = +75%; d2 = +25%* |  |
| 4 months | -4.14 (-7.15 to -1.13) |
| 8 months | -4.40 (-7.48 to -1.31) |
| 24 months | -4.56 (-8.23 to -0.90) |
| SDI – *d1 = +75%; d2 = +50%* |  |
| 4 months | -4.07 (-7.17 to -0.97) |
| 8 months | -4.33 (-7.50 to -1.16) |
| 24 months | -4.38 (-8.11 to -0.64) |
| SDI – *d1 = +75%; d2 = +75%* |  |
| 4 months | -4.01 (-7.21 to -0.80) |
| 8 months | -4.26 (-7.54 to -0.98) |
| 24 months | -4.18 (-8.00 to -0.36) |
| SDI – *d1 = +75%; d2 = -25%* |  |
| 4 months | -4.26 (-7.15 to -1.38) |
| 8 months | -4.52 (-7.49 to -1.56) |
| 24 months | -4.92 (-8.48 to -1.37) |
| SDI – *d1 = +75%; d2 = -50%* |  |
| 4 months | -4.32 (-7.17 to -1.46) |
| 8 months | -4.58 (-7.51 to -1.66) |
| 24 months | -5.10 (-8.61 to -1.59) |
| SDI – *d1 = +75%; d2 = -75%* |  |
| 4 months | -4.37 (-7.20 to -1.53) |
| 8 months | -4.63 (-7.53 to -1.73) |
| 24 months | -5.26 (-8.73 to -1.79) |

## Analyses of SDI at 24 months (estimand B)

The secondary estimand of interest is obtained from a 2-level mixed effects model of the 24 months SDI score allowing for clustering by facilitator in the intervention arm. The model included a treatment group indicator, baseline SDI score and indicators for site as fixed effects. Measurements at 4 and 8 months were not included in this analysis.

Table 7: Results of analysis for estimand B

|  | Treatment as usual | | Intervention | | Adjusted mean difference (95% CI) |
| --- | --- | --- | --- | --- | --- |
|  | n | Mean (SD) | n | Mean (SD) |  |
| SDI |  |  |  |  |  |
| 24 months | 84 | 19.2 (14.7) | 92 | 14.4 (11.4) | -5.51 (-9.45 to -1.58) |

An additional analysis to adjust for variables that showed some imbalance between groups at baseline (PLWD sex, carer sex and relationship to carer) was carried out, and the results are similar; -5.31 (-9.33 to -1.30)

**Sensitivity analyses for missing outcome (estimand B)**

We used the multiple imputation data generated in Section 1.2 to carry out sensitivity analyses for missing outcomes for estimand B.

The main analysis model was fitted for each imputed data set and the estimates were combined using Rubin’s rules. This was done using *mi estimate* function in Stata, which does not support the Kenward-Roger method for adjusted degrees of freedom.

Table 8: Results of multiple imputation analysis of the estimand B

|  | Adjusted mean difference (95% CI) |
| --- | --- |
|  |  |
| SDI |  |
| 24 months | -4.82 (-8.54 to -1.09) |

*Pattern mixture models under MNAR assumption*

We followed the same approach as described in Section 2.3 to fit pattern mixture models under MNAR.

Table 9: Results of pattern mixture models under MNAR assumption for varying values of *d1* and *d2* for estimand B.

|  | Adjusted mean difference (95% CI) |
| --- | --- |
| SDI – *d1 = +25%; d2 = +25%* |  |
| 24 months | -4.58 (-8.47 to -0.69) |
| SDI – *d1 = +25%; d2 = +50%* |  |
| 24 months | -4.36 (-8.43 to -0.29) |
| SDI – *d1 = +25%; d2 = +75%* |  |
| 24 months | -4.13 (-8.40 to 0.14) |
| SDI – *d1 = +25%; d2 = -25%* |  |
| 24 months | -5.00 (-8.62 to -1.39) |
| SDI – *d1 = +25%; d2 = -50%* |  |
| 24 months | Did not converge |
| SDI – *d1 = +25%; d2 = -75%* |  |
| 24 months | -5.36 (-8.80 to -1.93) |
| SDI – *d1 = +50%; d2 = +25%* |  |
| 24 months | -4.55 (-8.48 to -0.63) |
| SDI – *d1 = +50%; d2 = +50%* |  |
| 24 months | -4.33 (-8.43 to -0.23) |
| SDI – *d1 = +50%; d2 = +75%* |  |
| 24 months | -4.10 (-8.39 to 0.20) |
| SDI – *d1 = +50%; d2 = -25%* |  |
| 24 months | -4.98 (-8.63 to -1.33) |
| SDI – *d1 = +50%; d2 = -50%* |  |
| 24 months | Did not converge |
| SDI – *d1 = +50%; d2 = -75%* |  |
| 24 months | -5.35 (-8.82 to -1.88) |
| SDI – *d1 = +75%; d2 = +25%* |  |
| 24 months | -4.53 (-8.49 to -0.56) |
| SDI – *d1 = +75%; d2 = +50%* |  |
| 24 months | -4.30 (-8.44 to -0.17) |
| SDI – *d1 = +75%; d2 = +75%* |  |
| 24 months | -4.06 (-8.40 to 0.27) |
| SDI – *d1 = +75%; d2 = -25%* |  |
| 24 months | -4.96 (-8.65 to -1.27) |
| SDI – *d1 = +75%; d2 = -50%* |  |
| 24 months | -5.16 (-8.75 to -1.57) |
| SDI – *d1 = +75%; d2 = -75%* |  |
|  | Did not converge |

# Analysis of secondary outcomes

## Binary secondary outcome: psychotropic medication

Table 10: Summary of use of hypnotics and anxiolytics medications, including melatonin.

|  | Treatment as usual (n=189) | Intervention (n=188) |
| --- | --- | --- |
| Baseline |  |  |
| No | 144 (76.2%) | 138 (73.4%) |
| Yes | 45 (23.8%) | 50 (26.6%) |
| 4-month |  |  |
| No | 126 (72.8%) | 119 (71.3%) |
| Yes | 47 (27.2%) | 48 (28.7%) |
| Missing | 16 | 21 |
| 8-month |  |  |
| No | 115 (70.6%) | 111 (69.8%) |
| Yes | 48 (29.4%) | 48 (30.2%) |
| Missing | 26 | 29 |
| 24-month |  |  |
| No | 60 (70.6%) | 64 (69.6%) |
| Yes | 25 (29.4%) | 28 (30.4%) |
| Missing | 104 | 96 |

Table 11: Summary of use of antidepressant medications

|  | Treatment as usual (n=189) | Intervention (n=188) |
| --- | --- | --- |
| Baseline |  |  |
| No | 142 (75.1%) | 143 (76.1%) |
| Yes | 47 (24.9%) | 45 (23.9%) |
| 4-month |  |  |
| No | 133 (76.9%) | 126 (75.4%) |
| Yes | 40 (23.1%) | 41 (24.6%) |
| Missing | 16 | 21 |
| 8-month |  |  |
| No | 126 (77.3%) | 124 (78.0%) |
| Yes | 37 (22.7%) | 35 (22.0%) |
| Missing | 26 | 29 |
| 24-month |  |  |
| No | 56 (65.9%) | 65 (70.7%) |
| Yes | 29 (34.1%) | 27 (29.3%) |
| Missing | 104 | 96 |

Table 12: Summary of use of antipsychotic medications

|  | Treatment as usual (n=189) | Intervention (n=188) |
| --- | --- | --- |
| Baseline |  |  |
| No | 177 (93.7%) | 174 (92.6%) |
| Yes | 12 (6.3%) | 14 (7.4%) |
| 4-month |  |  |
| No | 155 (89.6%) | 151 (90.4%) |
| Yes | 18 (10.4%) | 16 (9.6%) |
| Missing | 16 | 21 |
| 8-month |  |  |
| No | 145 (89.0%) | 142 (89.3%) |
| Yes | 18 (11.0%) | 17 (10.7%) |
| Missing | 26 | 29 |
| 24-month |  |  |
| No | 76 (89.4%) | 80 (87.0%) |
| Yes | 9 (10.6%) | 12 (13.0%) |
| Missing | 104 | 96 |

Table 13: Summary of use of at least one psychotropic medication.

|  | Treatment as usual (n=189) | Intervention (n=188) |
| --- | --- | --- |
| Baseline |  |  |
| No | 107 (56.6%) | 100 (53.2%) |
| Yes | 82 (43.4%) | 88 (46.8%) |
| 4-month |  |  |
| No | 96 (55.5%) | 86 (51.5%) |
| Yes | 77 (44.5%) | 81 (48.5%) |
| Missing | 16 | 21 |
| 8-month |  |  |
| No | 85 (52.1%) | 87 (54.7%) |
| Yes | 78 (47.9%) | 72 (45.3%) |
| Missing | 26 | 29 |
| 24-month |  |  |
| No | 38 (44.7%) | 43 (46.7%) |
| Yes | 47 (55.3%) | 49 (53.3%) |
| Missing | 104 | 96 |

*Regression modelling of the use of at least one psychotropic medication*

The mixed effects binomial generalised linear model with identity link did not converge, even when clustering due to facilitator was removed. As such, a binomial generalised linear model with link identity is fitted using the 24-month data as outcome to estimate the risk difference and the standard error computed to allow for facilitator clustering. This model adjusted for baseline use of psychotropic medication only, when site was included in the model, convergence was not achieved.

Table 14: Results of analysis of use of psychotropic medication to estimate the risk difference.

|  | Treatment as usual | Intervention | Adjusted risk difference (95% CI)* |
| --- | --- | --- | --- |
|  | N (%) | N (%) |  |
| Use of at least one psychotropic medication |  |  |  |
| 24 months | 47 (55.3%) | 49 (53.3%) | -0.04 (-0.16 to 0.09) |

* Estimated from a binomial generalised linear model with identity link adjusting for baseline use of psychotropic medication, N = 177.

Table 14: Results of analysis of use of psychotropic medication to estimate the odds ratio.

|  | Treatment as usual | Intervention | Adjusted odds ratio (95% CI)* |
| --- | --- | --- | --- |
|  | N (%) | N (%) |  |
| Use of at least one psychotropic medication |  |  |  |
| Baseline | 82 (43.4%) | 88 (46.8%) | - |
| 4 months | 77 (44.5%) | 81 (48.5%) | 1.26 (0.57 to 2.76) |
| 8 months | 78 (47.9%) | 72 (45.3%) | 0.71 (0.32 to 1.59) |
| 24 months | 47 (55.3%) | 49 (53.3%) | 0.61 (0.22 to 1.74) |

* Estimated from a mixed effects binomial generalised linear model with logit link adjusting for site and baseline use of psychotropic medication as fixed effects and accounting for clustering by facilitator and participant, N = 346.

## Side effects

Table 15: Side effects at 24-months

|  | Treatment as usual (n=85) | Intervention (n=92) |
| --- | --- | --- |
|  | N (%) | N (%) |
| Falls- yes | 36 (42.2%) | 39 (42.4%) |
| Mild | 11 (30.6%) | 14 (35.9%) |
| Moderate | 13 (36.1%) | 12 (30.8%) |
| Severe | 12 (33.3%) | 13 (33.3%) |
|  |  |  |
| Gastroinstestinal -yes | 35 (41.2%) | 61 (66.3%) |
| Mild | 7 (20.0%) | 10 (16.4%) |
| Moderate | 28 (80.0%) | 47 (77.0%) |
| Severe | 0 (0%) | 4 (6.6%) |
|  |  |  |
| Neurological – yes | 58 (68.2%) | 52 (56.5%) |
| Mild | 8 (13.8%) | 8 (15.4%) |
| Moderate | 47 (81.0%) | 41 (78.8%) |
| Severe | 3 (5.2%) | 3 (5.8%) |
|  |  |  |
| Infections – yes | 29 (34.1%) | 45 (48.9%) |
| Mild | 1 (3.4%) | 6 (13.3%) |
| Moderate | 22 (75.9%) | 28 (62.2%) |
| Severe | 6 (20.7%) | 11 (24.4%) |
|  |  |  |
| Other side effect - yes | 53 (62.4%) | 53 (57.6%) |

## Survival type secondary outcomes

Table 16: Proportions of PLWD died or moved to care home.

|  | Treatment as usual (n=189) | Intervention (n=188) |
| --- | --- | --- |
| 4-month |  |  |
| PLWD deceased | 9 (4.8%) | 9 (4.8%) |
| PLWD moved to care home | 8 (4.2%) | 4 (2.1%) |
| TOTAL | 17 (9.0%) | 13 (6.9%) |
| 8-month |  |  |
| PLWD deceased | 17 (9.7%) | 17 (10.0%) |
| PLWD moved to care home | 14 (8.0%) | 11 (6.5%) |
| TOTAL | 31 (17.6%) | 28 (16.5%) |
| 24-month |  |  |
| PLWD deceased | 47 (28.8%) | 49 (30.8%) |
| PLWD moved to care home | 34 (20.9%) | 37 (23.3%) |
| TOTAL | 79 (48.5%) | 79 (49.7%) |

Figure1: Kaplan-Meier plot for probability of death

Figure 2: Kaplan-Meier plot for probability of care home admission

Figure3: Kaplan-Meier plot for probability of death or care home admission (using care home admission date if moved prior to death)

Time to admission to care home and death are formally compared using a parametric (Weibull) shared frailty model, that allows for facilitator clustering in the intervention arm and adjusts for intervention arm and study site as a fixed effect.

Table 17: Results of parametric shared frailty model for time to admission to care home, time to death, and the composite (whichever event came first)

|  | Hazard ratio (95% CI) |
| --- | --- |
|  |  |
| Time to death | 1.05 (0.64 to 1.74) |
| Time to care home admission | 1.11 (0.67 to 1.83) |
| Time to death or care home admission | 1.02 (0.70 to 1.50) |

### Supportive analysis

A competing risks model was fitted for time to death and time to care home admission. The models adjust for intervention arm and study site. The models report the sub hazard ratio indicating the hazard ratio of the event occurring in the presence of a competing event.

|  | Sub-hazard ratio (95% CI) |
| --- | --- |
|  |  |
| Time to death | 1.01 (0.61 to 1.67) |
| Time to care home admission | 1.12 (0.68 to 1.86) |
